# Regulation of phagocyte triglyceride by a STAT-ATG2 pathway controls mycobacterial infection

**DOI:** 10.1038/ncomms14642

**Published:** 2017-03-06

**Authors:** Claire B. Péan, Mark Schiebler, Sharon W. S. Tan, Jessica A. Sharrock, Katrin Kierdorf, Karen P. Brown, M. Charlotte Maserumule, Shinelle Menezes, Martina Pilátová, Kévin Bronda, Pierre Guermonprez, Brian M. Stramer, R. Andres Floto, Marc S. Dionne

**Affiliations:** 1 MRC Centre for Molecular Bacteriology and Infection, and Department of Life Sciences, Imperial College London, Ground Floor, Flowers Building, South Kensington Campus, London SW7 2AZ, UK; 2 Molecular Immunity Unit, MRC Laboratory of Molecular Biology, University of Cambridge Department of Medicine, Cambridge CB2 0QH, UK; 3 Centre for Molecular and Cellular Biology of Inflammation, School of Medicine, King's College London, London SE1 1UL, UK; 4 Cambridge Centre for Lung Infection, Papworth Hospital, Cambridge CB23 3RE, UK; 5 Randall Division of Cell and Molecular Biophysics, King's College London, London SE1 1UL, UK

## Abstract

*Mycobacterium tuberculosis* remains a global threat to human health, yet the molecular mechanisms regulating immunity remain poorly understood. Cytokines can promote or inhibit mycobacterial survival inside macrophages and the underlying mechanisms represent potential targets for host-directed therapies. Here we show that cytokine-STAT signalling promotes mycobacterial survival within macrophages by deregulating lipid droplets via ATG2 repression. In *Drosophila* infected with *Mycobacterium marinum*, mycobacterium-induced STAT activity triggered by *unpaired*-family cytokines reduces *Atg2* expression, permitting deregulation of lipid droplets. Increased *Atg2* expression or reduced macrophage triglyceride biosynthesis, normalizes lipid deposition in infected phagocytes and reduces numbers of viable intracellular mycobacteria. In human macrophages, addition of IL-6 promotes mycobacterial survival and BCG-induced lipid accumulation by a similar, but probably not identical, mechanism. Our results reveal *Atg2* regulation as a mechanism by which cytokines can control lipid droplet homeostasis and consequently resistance to mycobacterial infection in *Drosophila*.

## Abstract (web summary)

Cytokines and their associated pathways can affect survival of *Mycobacterium tuberculosis* in macrophages, representing potential targets for host-directed therapies. Here, Péan *et al*. show that cytokine-STAT signalling promotes mycobacterial survival within macrophages by deregulating lipid droplet homeostasis.

Virulent mycobacteria are critically dependent on their ability to survive within macrophages to cause disease. The tendency of macrophages to support or kill mycobacteria is directly regulated by distinct cytokine–JAK–STAT pathways: for example, the Th1 cytokine interferon-γimproves the ability of the macrophage to kill mycobacteria, whereas interleukin (IL)-10 and the Th2-derived cytokines IL-4 and IL-13 can inhibit killing of intracellular mycobacteria^1^,^2^,^3^. Our knowledge of the *in vivo* functions of these signals in mycobacterial infection, and of their transcriptional targets and the associated physiologies, remains incomplete, especially for those signals that inhibit killing of intracellular bacteria^4^,^5^. The genetic and cell-biological mechanisms that underlie these responses are potentially fertile ground for host-targeted therapies for tuberculosis^6^,^7^.

The fruitfly *Drosophila melanogaster* is a well-established model for the study of innate immunity^8^. Many aspects of the innate immune response to microbes are conserved between flies and vertebrates, including the central role of nuclear factor-κB family transcription factors and Toll-like receptors, anti-inflammatory effects of transforming growth factor-βfamily signals and the presence of bactericidal phagocytes homologous to vertebrate macrophages^9^,^10^,^11^. We have developed a system in which *Drosophila* are infected with *Mycobacterium marinum*, which allows us to identify host mechanisms involved in mycobacterial infection^12^,^13^. As in mammals, cytokine production by phagocytes is a critical component of the cellular response to infection. Flies have three known genes encoding IL-like signals, *upd1*, *upd2* and *upd3* (ref. 14). These proteins have structural and functional similarity to the four-helix-bundle cytokines of mammals^15^. They bind a GP130-like receptor, encoded by *domeless*, to activate a conserved JAK–STAT signalling cassette^16^. Upon bacterial infection in adult flies, *upd3* is expressed by phagocytes and activates the JAK–STAT pathway in the fat body, where it is required for infection-induced expression of the stress peptide *TotA*^14^. This signalling pathway is critically required for antiviral defense^17^, it regulates larval haematopoiesis^18^ and it is important in maintaining gut integrity in response to bacterial infection and other stresses^19^,^20^,^21^,^22^,^23^. Other roles in immune defense against bacteria are unknown.

Here we show that *unpaired*-family cytokines are critical regulators of defense against intracellular virulent mycobacteria. We show that *M. marinum* infection drives production of *upd3* in phagocytes, and that reception of this signal by phagocytes is detrimental to the host. JAK–STAT blockade increased resistance to infection: it prolonged survival of the host, reduced mycobacterial numbers and delayed immune cell death, with similar effects observed *in vitro*. This effect was associated with overexpression of the autophagy-related gene *Atg2* and not other autophagy genes, *in vivo* and *in vitro*. *M. marinum* infection of cultured phagocytes increased intracellular neutral lipids and drove accumulation of unusually large lipid droplets (LDs). *Atg2* overexpression or JAK–STAT inhibition reduced intracellular mycobacterial number and partially normalized phagocyte lipid droplet size without significantly changing bulk autophagy. Direct inhibition of triglyceride synthesis prevented the infection-induced alteration in LDs, reduced mycobacteria–LD association and reduced intracellular viable mycobacteria. We then tested these findings in human cells. Similar to *Drosophila*, IL-6 signalling in macrophages increased intracellular Bacillus Calmette–Guérin (BCG) and *Mycobacterium tuberculosis* numbers in a dose-dependent manner; ATG2A expression was also reduced, although only to a very small extent. Importantly, the ability of IL-6 to increase the number of viable intracellular mycobacteria was abolished by inhibition of macrophage triglyceride synthesis. Together, these data indicate that loss of a STAT-activating cytokine pathway can reduce survival of intracellular mycobacteria via effects on cellular lipid deposition. In *Drosophila*, one responsible effector may be ATG2.

## Results

### *unpaired* signalling decreases resistance to *M. marinum*

**Figure 1:**
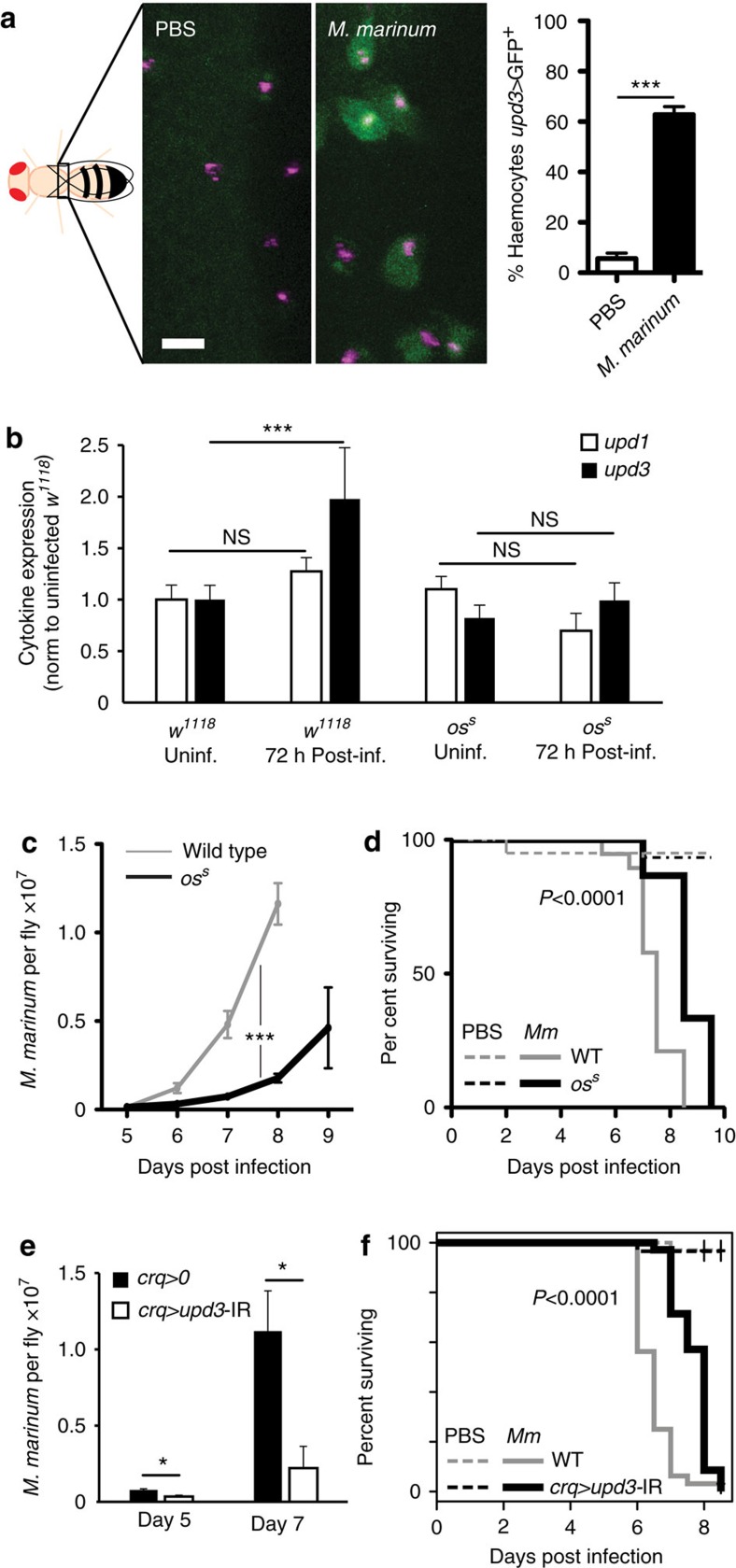
The *unpaired* cytokines reduce resistance to *M. marinum* infection. (**a**) *upd3* expression in haemocytes after *M. marinum* infection. Left, intravital imaging of eGFP expressed under the control of *upd3*-Gal4 in flies injected with PBS or *M. marinum* (5,000 CFU). Haemocytes are labelled with nuclear dsRed directly driven by the *HmlΔ* promoter^10^. This image was taken 48 h after injection. Scale bar, 10 μm. Right, quantification of the fraction of GFP-positive cells from seven flies based on images similar to the one shown. Error bars show s.d. Values are statistically different (***P<0.001) by Mann–Whitney test. (**b**) mRNA expression of *upd1* and *upd3* in wild-type (*w*^*1118*^) and *os*^*s*^ flies, uninfected or 72 h after infection with *M. marinum*. Assayed by qRT–PCR and normalized to *Rpl1* (as a loading control), and then to uninfected wild-type flies for each gene. *n*=4 for each condition. Data were log-transformed before statistical comparison by Student's *t*-test (***P<0.001). (**c**) *M. marinum* burden in wild-type and *os*^*s*^ mutant flies, assayed by qRT–PCR. Flies were injected with 500 CFU *M. marinum* and kept at 25 °C. *P*-value for differences in bacterial numbers by two-way analysis of variance (ANOVA), *n*=6 per time point except for day 9 where *n*=3 for *os*^*s*^ flies. (**d**) Survival of wild-type and *os*^*s*^-mutant flies, injected with PBS or infected with 500 CFUs *M. marinum*, as indicated. Flies were kept at 25 °C. *P*-value for difference between infected survival curves by log-rank test. (**e**) *M. marinum* burden in control (*w*^*1118*^;; *crq-Gal4*/+) and haemocyte-specific *upd3*-knockdown (*w*^*1118*^; UAS-*upd3*-IR/+; *crq-Gal4*/+) flies, assayed by qRT–PCR. Flies were injected with 500 CFU *M. marinum* and kept at 25 °C. Here and elsewhere, ‘*crq*>*upd3*-IR' is used as an abbreviation for ‘*crq*-Gal4, UAS-*upd3*-IR'. *P*-value for differences in bacterial numbers by one-way ANOVA, *n*=3 per time point (*P<0.05). (**f**) Survival of control (*w*^*1118*^;; *crq-Gal4*/+) and haemocyte-specific *upd3*-knockdown (*w*^*1118*^; UAS-*upd3*-IR/+; *crq-Gal4*/+) flies, injected with PBS or 500 CFU *M. marinum*. Flies were kept at 25 °C. *P*-value for difference between infected survival curves by log-rank test.

The IL-6-like cytokine *upd3* can be induced in haemocytes by infection with non-pathogenic bacteria; however, the ability of bacterial pathogens to induce its expression has not been previously explored^14^. This was particularly in question with *M. marinum*, which is not a strong agonist of known pattern-recognition pathways in *Drosophila*^12^. We thus examined the *in vivo* expression of *upd3* during the initial phase of *M. marinum* infection using an *upd3* green fluorescent protein (GFP) reporter and a haemocyte-specific dsRed nuclear marker^10^,^14^. We observed strong GFP induction in adult *Drosophila* haemocytes after mycobacterial infection (Fig. 1a), indicating that mycobacterial infection was indeed sufficient to drive *upd3* production from macrophages *in vivo*. This effect was also visible by quantitative reverse transcriptase–PCR (qRT–PCR) in samples from whole flies (Fig. 1b).

To test the role of *upd3* in mycobacterial infection in *Drosophila*, we infected flies carrying the *os*^*s*^ mutation with *M. marinum*. *os*^*s*^ affects expression of the cytokines *upd1* and *upd3*, and impairs induction of *upd3* by infection with non-pathogenic bacteria^14^,^24^. We observed that *os*^*s*^ mutants did not activate *upd3* expression to the same degree as wild-type flies after *M marinum* infection, whereas *upd1* expression was not induced by *M. marinum* infection in wild-type flies or *os*^*s*^ mutants (Fig. 1b). Despite identical starting inocula, *os*^*s*^ mutants carried a much lower mycobacterial burden at late stages of infection and survived longer than wild-type controls (Fig. 1c,d).

Although haemocytes were the only visible site of *upd3* induction in *M. marinum*-infected *upd3*>GFP flies, it remained possible that some other tissue was contributing to the relevant UPD3 pool. Moreover, although we saw no significant induction of *upd1* by infection, changes in UPD1 might still contribute to the *os*^*s*^ phenotype. To verify that haemocyte-derived UPD3 was responsible for the mycobacterial resistance phenotype of *os*^*s*^ mutants, we knocked down *upd3* with the haemocyte-specific *crq*-*Gal4* driver and examined mycobacterial pathogenesis^25^. Haemocyte *upd3* knockdown gave strong lifespan extension after infection and increased mycobacterial number (Fig. 1e,f). Together, these experiments indicate that haemocytes produce *upd3* upon mycobacterial infection and haemocyte-derived *upd3* shortens the infected lifespan of the host and impairs resistance against *M. marinum*.

### Haemocyte STAT activity decreases resistance to mycobacteria

To identify relevant UPD3 target tissues, we used RNA interference (RNAi) knockdown of *Stat92E* (the sole *Drosophila* STAT). Prior work has suggested that immune-induced STAT signalling is important in regulating humoral immune responses via effects on the *Drosophila* fat body^26^. However, *Stat92E* knockdown in the fat body had no effect on survival after *M. marinum* infection (Supplementary Fig. 1a). The primary direct immune effect of the fat body is the immune-inducible expression of antimicrobial peptides; we found that *os*^*s*^ mutant flies exhibited no consistent differences in antimicrobial peptide expression before haemocyte death (Supplementary Fig. 1b–e). We thus concluded that the effect of *upd3* on mycobacterial infection was independent of the fat body.

**Figure 2:**
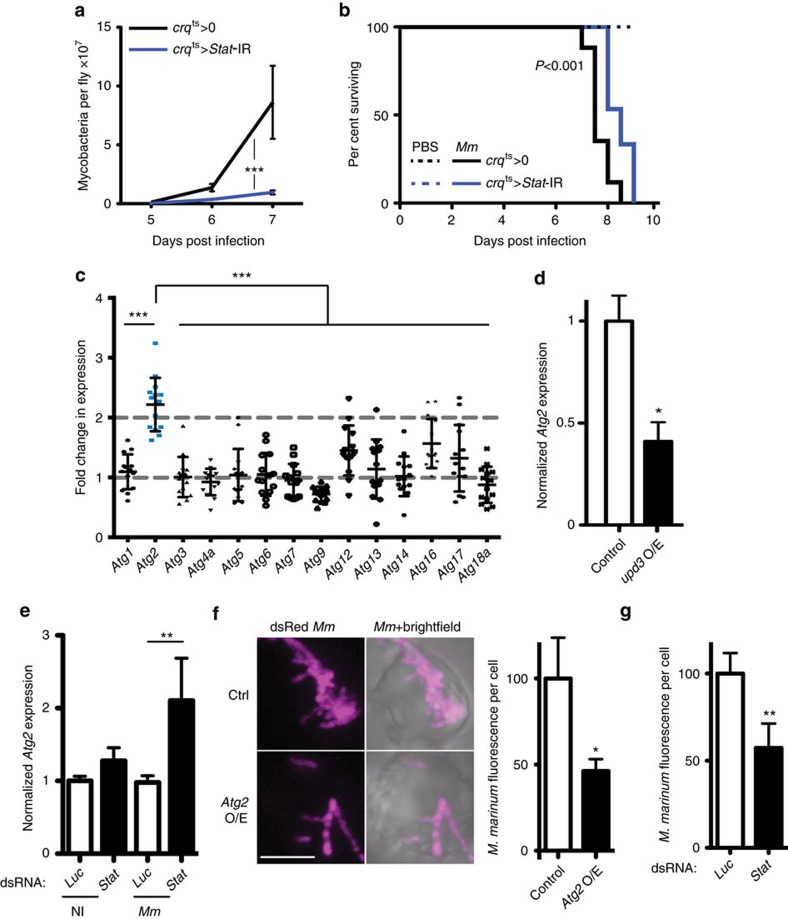
STAT activation in haemocytes impairs *Atg2* expression to reduce mycobactericidal activity. (**a**) *M. marinum* burden in control (*w*^*1118*^; *tub-Gal80*^*ts*^/*+*; *crq-Gal4*/+) and inducible haemocyte-specific *Stat92E*-knockdown (*w*^*1118*^, *UAS-Stat92E-IR*/*+; UAS-Stat92E-IR*/*tub-Gal80*^*ts*^*; crq-Gal4*/*+*) flies, assayed by qRT–PCR. Flies were initially infected with 500 CFU *M. marinum* and cultured at 29 °C. *P*-value for difference in bacterial numbers by two-way analysis of variance (ANOVA), *n*=5 per timepoint. (**b**) Survival of control (*w*^*1118*^*; tub-Gal80*^*ts*^/*+; crq-Gal4*/*+)* and haemocyte-specific *Stat92E*-knockdown (*w*^*1118*^, *UAS-Stat92E -IR*/*+; UAS-Stat92E -IR*/*tub-Gal80*^*ts*^*; crq-Gal4*/*+)* flies, injected with PBS or infected with 500 CFU *M. marinum*, as indicated. Infectious dose and temperature as in **a**. *P*-value for difference between infected survival curves by log-rank test. (**c**) Fold change in expression of several autophagy genes in *Stat92E*-IR *(w*^*1118*^*, UAS-Stat92E-IR; UAS-Stat92E-IR*/*tub-Gal80*^*ts*^*; crq-Gal4*/*+*) flies compared with controls (*w*^*1118*^; *tub-Gal80*^*ts*^/*+*; *crq-Gal4*/+and *w*^*1118*^*, UAS-Stat92E-IR; UAS-Stat92-IR*/*+*) 5 days after injection with 500 CFU *M. marinum*. The value shown is the geometric mean of two ratios (knockdown/driver-only control and knockdown/STAT-IR only control). *Atg2* fold change is statistically different from other autophagy genes (***P<0.001 by Mann–Whitney test, *n*=14). (**d**) *Atg2* expression by qRT–PCR in S2R^+^ cells overexpressing *HA* (control) or *upd3*. Normalized to *Rpl1.* Values are statistically different (*P<0.05 by Mann–Whitney test, *n*=6). (**e**) Expression of *Atg2* by qRT–PCR in S2R+ cells with *Luciferase* or *Stat92E* knocked down, with or without *M. marinum* infection. Normalized to *Rpl1* and then to the uninfected *Luciferase* control. Values are statistically different as indicated (**P<0.01 by Mann–Whitney test, *n*=7). (**f**) Intracellular dsRed-expressing *M. marinum* in S2R^+^ cells overexpressing *HA* (control) or *Atg2*. Picture is a representative; graph shows normalized *M. marinum* fluorescence from 37 (*Atg2* O/E) or 45 (control) cells. Values are statistically different (*P<0.05 by Mann–Whitney test). Scale bar, 5 μm. (**g**) Intracellular dsRed-expressing *M. marinum* in S2R^+^ cells with *Luciferase* or *Stat92E* knocked down by RNAi. The graph shows normalized *M. marinum* fluorescence from 44 (*Stat* dsRNA) or 76 (*Luc* dsRNA) cells. Values are statistically different (**P<0.01 by Mann–Whitney test).

We next tested the effect of cytokine signalling on haemocytes themselves. As the JAK–STAT pathway regulates larval haematopoiesis, we combined our haemocyte drivers with *Gal80*^*ts*^, making their effect temperature sensitive, to enable adult-specific *Stat92E* knockdown^18^,^27^,^28^. Adult-specific *Stat92E* knockdown or expression of a dominant-negative form of the *upd3* receptor *dome* in haemocytes significantly improved resistance to infection (Fig. 2a,b and Supplementary Fig. 2). These results together indicate that haemocyte-derived *upd3* activates STAT signalling in haemocytes, decreasing resistance to mycobacterial infection and impairing host survival.

Cell death via necrosis or apoptosis is commonly observed in vertebrate macrophages infected with *M. tuberculosis* or *M. marinum*^29^,^30^,^31^. We observed marked cytotoxicity due to *M. marinum* infection in *Drosophila* S2R^+^ cells, an embryonic-derived macrophage cell line, and progressive loss of haemocytes during the last few days of *M. marinum* infection *in vivo* (Supplementary Fig. 3a–d). Just before dying, flies harboured no visible haemocytes and dramatically reduced expression of the haemocyte marker *Hml* was detected by qRT–PCR on the whole fly (Supplementary Fig. 3c–e). The *os*^*s*^ mutation improved haemocyte survival in infected flies (Supplementary Fig. 3b–d). Importantly, this was not a result of differential microbial internalization, as the fraction of haemocytes containing *M. marinum* was not different between control and *upd3-IR* flies 16 h after infection (Supplementary Fig. 4a,b). The difference in haemocyte survival, such as the difference in haemocyte number, was also seen in flies with adult-specific haemocyte *Stat92E* knockdown (Supplementary Fig. 4c–e). We thus conclude that *upd3-STAT* signalling alters the interaction between mycobacteria and phagocytes so as to impair the survival of infected cells.

### JAK–STAT activation in haemocytes downregulates *Atg2*

We wished to identify target genes and cell-biological processes behind the reduced mycobacterial numbers seen as a result of impaired cytokine signalling. Activation of JAK–STAT signalling in Kc167 cells reduces expression of several autophagy-related genes^32^. As phagocyte autophagy can impair intracellular survival of *M. tuberculosis*^33^, we tested this transcriptional effect *in vivo*, analysing the expression levels of 14 autophagy-related genes in flies with haemocyte-specific *Stat92E* knockdown. Surprisingly, *Atg2* was the only autophagy gene with strongly enhanced expression following mycobacterial infection in *Stat92E* knockdown flies (Fig. 2c). Further analysis revealed that *Atg2* overexpression was seen in haemocyte-specific *Stat92E* and *upd3* knockdown flies independent of infection (Supplementary Fig. 5a,b). Conversely, *in vitro*, *upd3* overexpression induced the known JAK–STAT pathway target *TotA* and reduced *Atg2* expression (Fig. 2d and Supplementary Fig. 5c,d). The specific effect on *Atg2* expression was particularly interesting, as in HeLa cells *Atg2* is separably important for autophagy and neutral lipid distribution, both processes associated with intracellular survival or killing of mycobacteria^33^,^34^,^35^,^36^.

To clarify how cytokines interacted with other inflammatory cues to regulate phagocyte *Atg2*, we measured expression of *Atg2* in S2R^+^ cells with or without *Stat92E* knockdown and with or without *M. marinum* infection. *Stat92E* knockdown or *M. marinum* infection gave mild or no increase in *Atg2* expression but, when combined, these two stimuli strongly induced *Atg2* expression, suggesting that *upd3* limits infection-induced *Atg2* overexpression (Fig. 2e). The difference observed between the *in vivo* context (where infection is not required for increased *Atg2* expression in *Stat92E* knockdowns) and these *in vitro* observations may be the result of the different cytokine milieu experienced by cells in culture, compared with those in the animal, or to the contribution of other tissues.

### Increased *Atg2* impairs intracellular *M. marinum* survival

As *Atg2* was specifically affected by loss of *upd3-Stat92E* signalling, we assayed the effect of *Atg2* overexpression in cultured phagocytes. Overexpression of GFP-ATG2 or RNAi of *Stat92E* reduced by 50% the number of intracellular mycobacteria per cell and eliminated mycobacterium-induced phagocyte death (Fig. 2f,g and Supplementary Fig. 5e,f). The effect of *Atg2* on resistance to infection was not due to general changes in particle internalization, as *Atg2* overexpression did not impair uptake of pHrodo-labelled beads (Supplementary Fig. 5g).

*Atg2* knockdown has separable effects on autophagy and lipid deposition in HeLa cells^35^. We assayed the effect of *Atg2* overexpression on autophagy and found no clear differences in autophagosome number, processing of ATG8 or association of autophagosomes with mycobacteria (Supplementary Fig. 6).

### *M. marinum* benefits from neutral lipids in phagocytes

**Figure 3:**
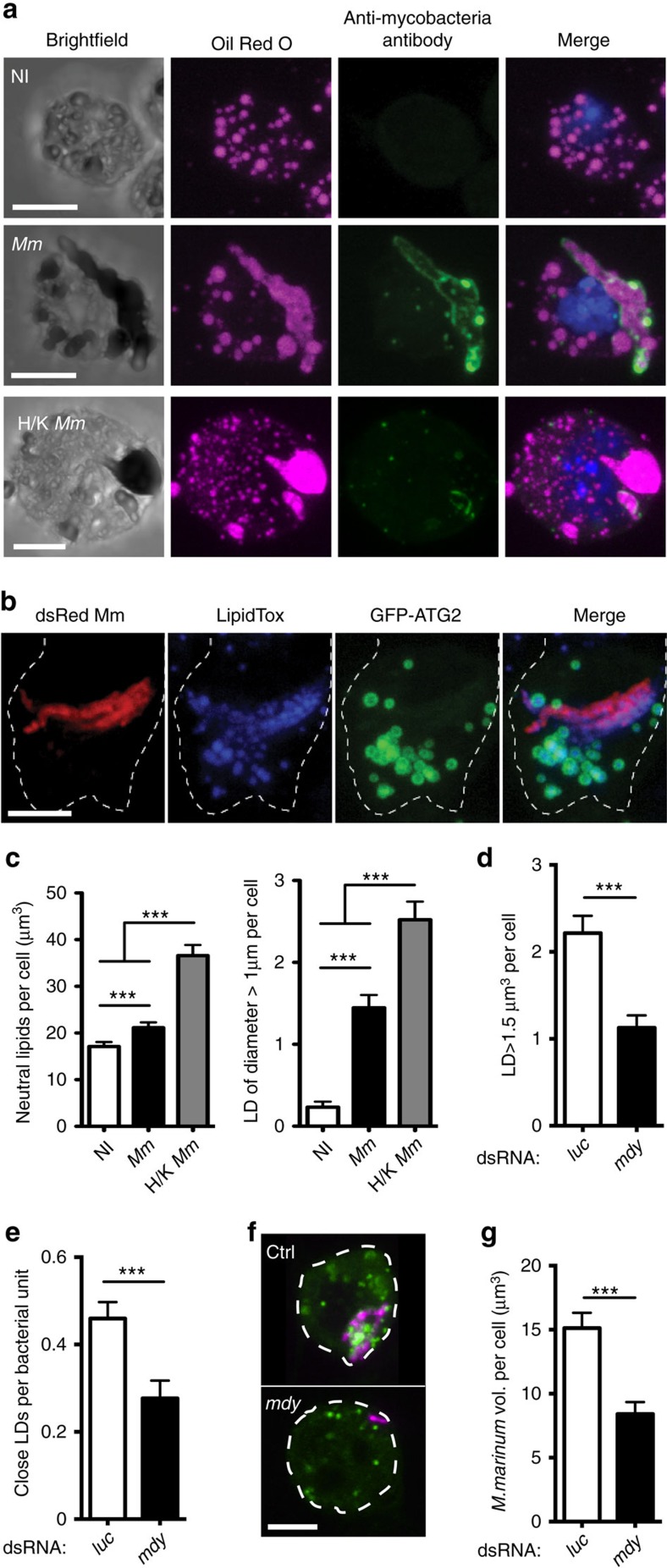
*M. marinum* driven lipid accumulation is required for intracellular growth or survival of mycobacteria. (**a**) Neutral lipids (stained with Oil Red O) and intracellular mycobacteria (stained with fluorescein isothiocyanate-anti-*M tuberculosis*) in cells variously uninfected, infected with live *M. marinum* or infected with heat-killed (H/K) *M. marinum*. (**b**) Co-localization of ATG2-GFP with LipidTox-stained intracellular neutral lipids. Cells were observed 48 h after transfection. Scale bars, 5 μm. (**c**) Quantification of neutral lipid volume per cell and total number of LDs with at least one axis >1 μm in length in cells treated as in **b**. Values are statistically different (**P<0.01 and ***P<0.001) by Mann–Whitney test. *n*=130 for non-infected cells, *n*=110 for *M.m*.-infected cells and *n*=96 for cells in contact with heat-killed *M.m.* (**d**) Number of LDs (stained with BODIPY 500/510) of volume ≥1.5 μm^3^ in cells treated with dsRNA for *mdy* or *Luc*, and infected with dsRed *M. marinum.* Values are statistically different (***P<0.001 by Mann–Whitney test, *n*=71 for *mdy* dsRNA, *n*=135 for *Luc* dsRNA). (**e**) Number of LDs (stained with BODIPY 500/510) at a distance ≤1 μm from bacteria and measured per unit of bacterial volume. Cells were treated with dsRNA for *mdy* or *Luc* and infected with dsRed *M. marinum*. Values are statistically different (***P<0.001 by Mann–Whitney test, *n*=64 for *mdy* dsRNA, *n*=119 for *Luc* dsRNA). (**f**) Co-localization of BODIPY-stained LDs with dsRed *M. marinum* in S2R^+^ cells with or without *mdy* knockdown. Scale bar, 5 μm. (**g**) Quantification of bacterial volume per cells in S2R^+^ cells treated with dsRNA for *mdy* or *Luc* and infected with dsRed *M. marinum*. Values are statistically different (***P<0.001 by Mann–Whitney test, *n*=64 for *mdy* dsRNA, *n*=119 for *Luc* dsRNA).

In *M. tuberculosis* infection, some macrophages accumulate LDs, which has been suggested to promote mycobacterial persistence within the host^37^,^38^. Indeed, intracellular survival of *M. tuberculosis* depends on its ability to recalibrate macrophage lipid homeostasis^34^,^39^. *M. marinum* infection, similar to *M. tuberculosis* infection, induces lipid accumulation in *Drosophila* cells (Fig. 3a). As we had seen little effect on general autophagy from *Atg2* overexpression, we explored the interaction of *Atg2* with LDs. GFP-ATG2 associated with many LDs, as previously seen in HeLa cells, and sometimes appeared to coat intracellular mycobacteria (Fig. 3b and Supplementary Fig. 6a)^35^.

Quantitative analysis of lipid deposition in infected cells revealed a mild increase in total intracellular neutral lipid, but a dramatic increase in the number of unusually large LDs in infected samples (Fig. 3c). Heat-killed *M. marinum* induced greater accumulation of LDs and more large lipid structures than live bacteria (Fig. 3c), whereas infection of S2R^+^ cells with *Escherichia coli*, as an activator of general inflammatory signals, did not alter neutral lipids (Supplementary Fig. 8a,b). Although we were able to stain neutral lipids within free mycobacteria, the size and shape of the lipid inclusions we observed in infected phagocytes were incompatible with the lipids being exclusively intrabacterial and the large irregular lipid inclusions we observed were often but not always physically associated with intracellular mycobacteria as detected by immunofluorescence or fluorescent protein expression (Fig. 3a,b and Supplementary Fig. 8c–e).

The fact that dead mycobacteria drove lipid accumulation and that large lipid inclusions were only partially associated with live intracellular *M. marinum* implied that these aberrant large lipid inclusions were host-derived and resulted from some change in host cell lipid deposition or metabolism exploited by mycobacteria. We tested this directly by RNAi inactivation of *midway* (*mdy*), the *Drosophila* homologue of diacylglycerol *O*-acyltransferase 1 (DGAT1), the final enzyme in triglyceride synthesis^40^. *mdy* knockdown reduced LD numbers and volume in uninfected and *M. marinum*-infected S2R+ cells (Supplementary Fig. 9a–f). We identified a threshold LD volume associated with pathogenesis by determining the cumulative distribution function of LD volumes in uninfected and *M marinum*-infected cells. These functions intersected at a volume of 1.5 μm^3^, indicating that most droplets of volume greater than this in infected cells were due to mycobacterial activity (Supplementary Fig. 7g). *mdy* knockdown impaired *M. marinum*-induced formation of large LDs and reduced mycobacterial association with LDs, leading to a general reduction in bacterial burden per cell (Fig. 3d–g). These data demonstrate that *M. marinum* survival and/or proliferation is dependent on lipid accumulation in *Drosophila* phagocytes.

### Increased *Atg2* expression reduces large LDs

**Figure 4:**
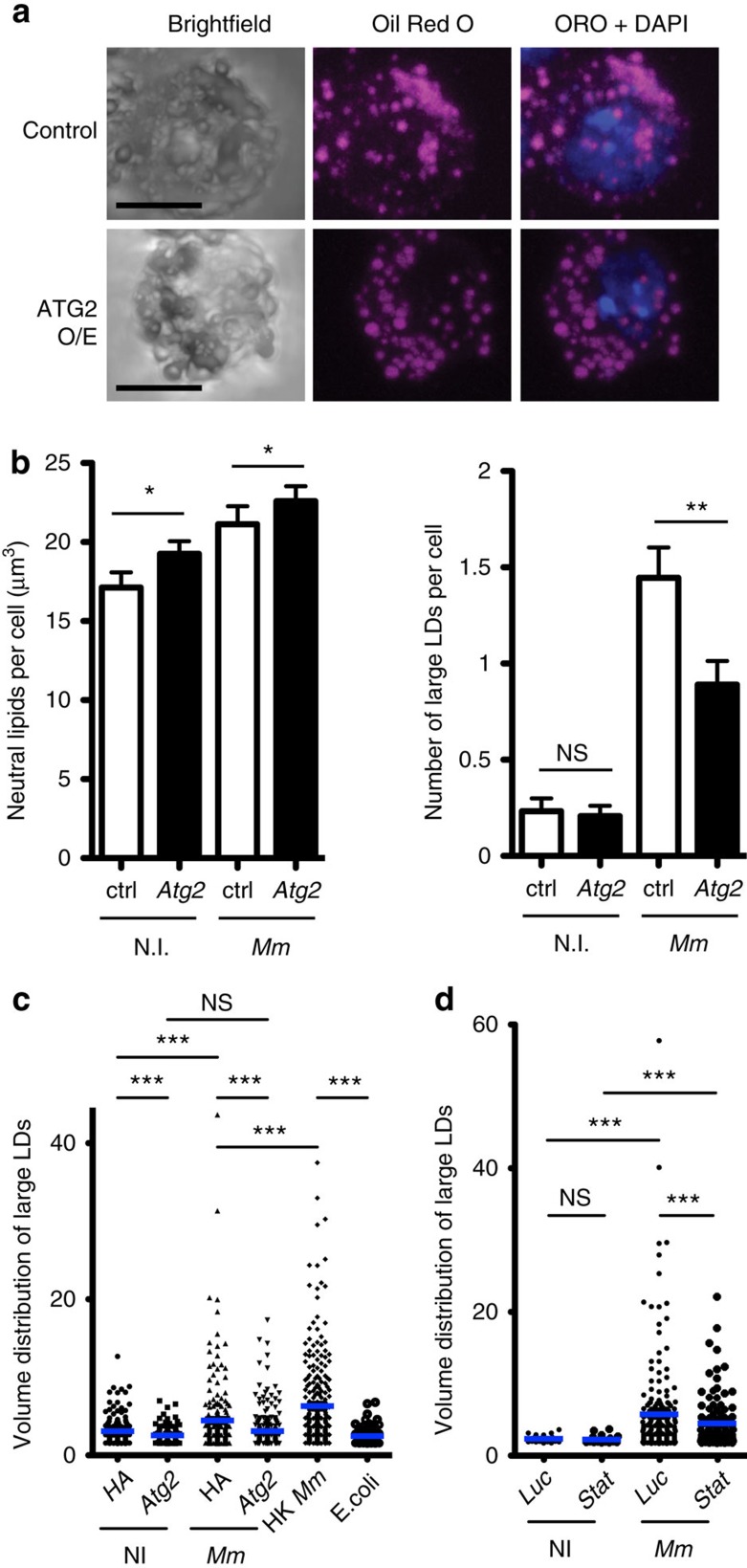
*Atg2* overexpression corrects LD size in *M. marinum*-infected cells. (**a**) Intracellular neutral lipid in *M. marinum*-infected cells overexpressing either *HA* (control) or *Atg2*. Scale bars, 5 μm. (**b**) Quantification of neutral lipid volume per cell and total number of LDs with at least one axis >1 μm in length in cells treated as in **a**. A slight increase in the overall volume of neutral lipids was observed in *Atg2*-overexpressing cells compared with control (*P<0.05 and **P<0.01, Mann–Whitney test, *n*=130 for non-infected controls, *n*=110 for Mm-infected controls, *n*=107 for non-infected cells overexpressing *Atg2* and *n*=100 for infected cells overexpressing *Atg2*). (**c**) Distribution of large (>1.5 μm^3^) LD volumes in cells overexpressing *Atg2* or *HA* and with the infections indicated. Volumes <1.5 μm^3^ are not shown but were included in statistical analysis. Punctae (3,100–5,000) were analysed depending on genotype and condition. Significance values are from the Kolmogorov–Smirnov test. (**d**) Distribution of large (>1.5 μm^3^) LD volumes in cells with *Luciferase* or *Stat92E* knocked down by RNAi, with or without infection as indicated. Volumes <1.5 μm^3^ are not shown but were included in statistical analysis. Punctae (1,100 to 2,000) were analysed depending on genotype and condition. Significance values are from the Kolmogorov–Smirnov test.

As preventing LD accumulation during infection reduced mycobacterial numbers and as ATG2 associated with LDs and reduced bacterial load, we hypothesized that ATG2 affected resistance to *M. marinum* by altering lipid deposition in immune cells. We tested this by infecting *Atg2*-transfected and control cells with *M. marinum* and observing LD volume (Fig. 4a–c). *M. marinum*-infected cells overexpressing *Atg2* exhibited a marked reduction in the number of large LDs compared with infected controls. *Stat92E* knockdown, which induced *Atg2* expression (Fig. 2e), also decreased large LD size in infected cells (Fig. 4d). *Stat92E* knockdown did not affect LD size in uninfected cells, in keeping with our observation that this knockdown did not affect *Atg2* expression in the absence of infection (Fig. 2e). These data together suggest that overexpression of *Atg2* observed in *upd3/Stat92E* knockdown flies renders phagocytes inhospitable to mycobacteria via effects on lipid deposition.

### IL-6 impairs mycobacterial killing via effects on triglyceride

**Figure 5:**
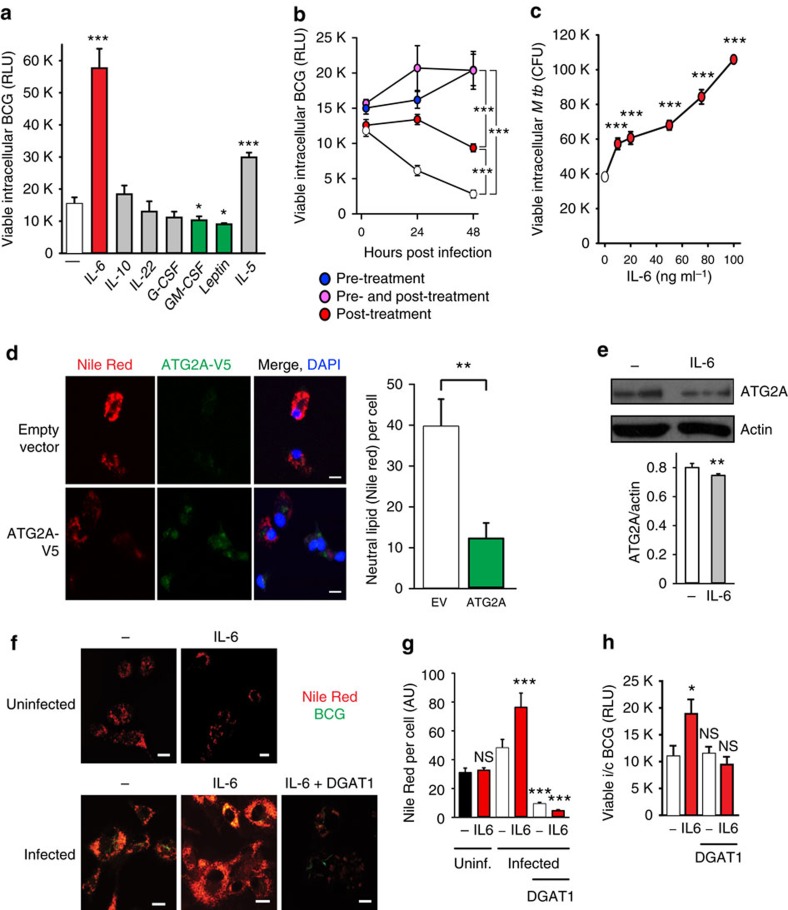
IL-6 impairs intracellular killing of *M. tuberculosis* through altered LD homeostasis. (**a**,**b**) Screening of the effect of cytokines signaling through JAK–STAT pathway on intracellular survival of mycobacteria in human macrophages. (**a**) Differentiated THP-1 cells were incubated with a luminescent strain of *M. bovis* BCG (BCG-lux) for 2 h, repeatedly washed, then treated with cytokines (20 ng ml^−1^) for 24 h at 37 °C. Cells were then lysed and luminescence measured. Viable intracellular mycobacteria were increased by IL-6 (red) and IL-5, and reduced by granuloyte–macrophage colony-stimulating factor and Leptin (green). Significance comparisons are between indicated treatment and untreated control. (**b**) Comparison of effects of treatment with IL-6 before and after infection in THP-1 cells. (**c**) Primary human macrophages were incubated with Bleupan *M. tuberculosis* for 2 h, repeatedly washed and then treated with IL-6 (red, at concentrations shown) for 24 h at 37 °C before cells were lysed and plated to count CFUs. Significance comparisons are between indicated treatment and untreated control. (**d**) ATG2A-V5 overexpression reduces intracellular neutral lipids in THP-1 cells. Scale bars, 10 μm. (**e**) Treatment of primary human macrophages with IL-6 (80 ng ml^−1^ for 24 h) causes a small reduction in ATG2A protein abundance. Quantification is from three independent experiments. Error bars represent s.e.m. (**f**,**g**) BCG-induced LD accumulation in enhanced by IL-6 treatment. Representative images (**f**) and fluorescence quantification (**g**) of intracellular LDs (red), in primary human macrophages 24 h after infection with GFP-labelled *M. bovis* BCG (green). LDs are increased following infection, further increased in the presence of IL-6 treatment (80 ng ml^−1^; red) and significantly reduced by pretreatment with the DGAT1 inhibitor T863 (10 μM, 48 h). Scale bars, 10 μm (**f**). (**h**) The increase in intracellular viable *M. bovis* BCG in primary human macrophages treated with IL-6 (red; 80 ng ml^−1^ for 24 h post infection) is blocked by pretreatment with the DGAT1 inhibitor T863 (as above). *P<0.05, **P<0.005 and ***P<0.001. Figures are representative of at least three independent experiments with at least three replicates per data point.

To see whether our results were relevant to human mycobacterial disease, we tested the ability of several JAK–STAT activating cytokines to impair killing of *Mycobacterium bovis* BCG by THP-1 cells. IL-6, added 2 h after infection, strongly inhibited the mycobactericidal activity of these cells (Fig. 5a). To further clarify the timing of this effect, we measured viable intracellular BCG in cells treated before and/or after infection with IL-6; we observed that either pre-, post- or pre- and postinfection IL-6 caused a significant increase in viable intracellular BCG at 24 and 48 h (Fig. 5b). We also observed that IL-6 caused a dose-dependent increase in survival of an auxotrophic mutant of *M. tuberculosis* within primary human macrophages (Fig. 5c). We then tested the effect of ATG2A overexpression on intracellular lipid stores in THP-1 cells and observed that overexpression of ATG2A was sufficient to markedly reduce neutral lipid stores in these cells (Fig. 5d). *upd3* has been suggested to be an evolutionary homologue of IL-6, and IL-6 is inducible by *M. tuberculosis* and can prevent killing of intracellular mycobacteria^15^,^41^,^42^; this suggested that the mechanisms of IL-6 action on human macrophages might be similar to the effects of *upd3* in *Drosophila*.

We next tested the effect of IL-6 treatment on abundance of ATG2A protein. Although IL-6 treatment caused a statistically significant reduction in ATG2A protein abundance in monocyte-derived macrophages (*P*=0.0384, unpaired Student's *t*-test), this effect was extremely small (Fig. 5e). Thus, although the STAT-ATG2 transcriptional circuit may function in similar ways in human and fly macrophages, it is difficult to imagine that the effect of exogenous IL-6 on mycobacterial number is driven by reductions in total quantity of cellular ATG2A.

We then examined the effect of IL-6 on lipid accumulation in infected primary human macrophages. We treated macrophages with IL-6 with or without pharmacological inhibition of DGAT1. IL-6 treatment potentiated the increase in intracellular lipid deposition driven by mycobacterial infection (Fig. 5f,g). DGAT1 inhibition reduced intracellular triglyceride levels, eliminated the ability of mycobacteria to drive lipid accumulation and abolished the ability of IL-6 to promote intracellular mycobacterial survival (Fig. 5f–h), indicating that the ability of IL-6 to increase intracellular mycobacterial numbers depended on its ability to enhance bacterial access to host triglyceride.

## Discussion

Here we identify a molecular mechanism that can reduce mycobacterial viability in phagocytes and show how this mechanism is inhibited by cytokine signalling *in vivo*. We show that haemocyte-derived cytokine signals activate JAK–STAT signalling in haemocytes in the early phases of *M. marinum* infection. Inhibiting production or response to this signal in adult haemocytes results in enhanced resistance to mycobacterial infection. We then show that flies in which either *upd3* or JAK–STAT signalling is impaired in haemocytes exhibit increased expression of *Atg2,* but not other autophagy genes. *Atg2* overexpression is sufficient to reduce mycobacterial survival in cultured *Drosophila* phagocytes and to inhibit changes in neutral lipid metabolism observed in *M. marinum-*infected cells. In parallel, we demonstrate that disturbing pathogen-induced changes in triglyceride metabolism directly impairs bacterial survival. Finally, a similar mechanism appears to function in human macrophages in response to IL-6 signalling. Together, these data indicate that some STAT-activating cytokines increase intracellular mycobacterial survival via effects on triglyceride deposition, that ATG2 regulation is a potential effector and that inhibition of macrophage triglyceride accumulation may be a useful therapeutic approach in mycobacterial disease.

Mycobacterium-infected macrophages often show aberrant accumulation of neutral lipids^43^, but the biological importance of this event is only partly understood. Intracellular mycobacteria depend on host lipids as a source of nutrients^37^,^39^, but this is not necessarily the only important role of neutral lipids in promoting mycobacterial survival. The fact that we observe changes in mycobacterial numbers as a result of triglyceride perturbations as little as 24 h after infection implies that accumulation of triglycerides may also directly disrupt phagocyte killing mechanisms. In this context, it may be relevant that neutral lipids have recently been observed to be protective against oxidative stress in the developing *Drosophila* central nervous system^44^, suggesting that lipid accumulation in macrophages might protect intracellular bacteria from oxidative killing mechanisms. Nonetheless, this effect will clearly not be universal: in some contexts, LDs contain large amounts of free histones that can exert direct antibacterial properties^45^. Another possibility is that the effect on bacterial survival is due to changes in production of lipids used as signalling molecules, such as leukotrienes, lipoxins and other eicosanoids^46^. Although the roles of these molecules in *Drosophila* immune responses has not been characterized, they play critical roles in regulation of vertebrate responses to mycobacterial infection^47^ and it is not difficult to imagine that their production would be altered by the effects on triglyceride deposition we document here. In either case, our observations suggest the possibility that fusion of LDs with bacteria-containing vesicles is normally a productive aspect of the host defense that has been co-opted by some intracellular pathogens to promote their own nutrition and/or protect themselves from intracellular killing—although other explanations for these phenomena cannot be excluded.

Although autophagy can be altered by pro- and anti-inflammatory cytokines and has been argued to be important in clearance of intracellular *M. tuberculosis*^3^,^33^, the *in vivo* relevance of this effect is not clear^48^. The role of autophagy in *M. marinum* infection is similarly unclear, though cytosolic *M. marinum* can be sequestered in double-membraned vesicles in bone-marrow-derived macrophages independently of the classical autophagy pathway proteins ATG5 or LC3, suggesting an unusual autophagy-like mechanism may be involved in the response to this organism^49^. Our work suggests that the effects of autophagy on infection and sequestration of mycobacteria and the effects of mycobacteria on intracellular lipid inclusions may be fundamentally linked by common underlying molecular mechanisms. Effects ascribed to autophagic mechanisms may, in fact, be due to autophagy components—in the case of our work, ATG2—acting outside of the normal autophagy pathways to alter lipid accumulation. Future work, in particular to delineate the genetic requirements for lipophagy as opposed to general autophagy, will help resolve these effects in infection.

**Figure 6:**
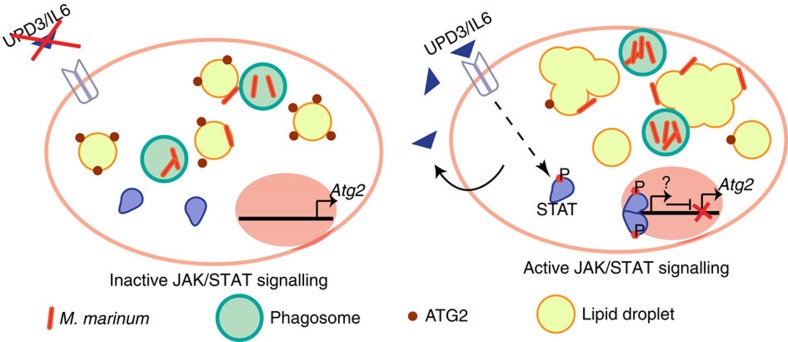
A model of the role of *upd3*-*Stat92E* signalling in the *M. marinum*-infected phagocyte. Upon infection, haemocytes secrete upd3, which activates the JAK–STAT pathway by signalling back to haemocytes. Upon activation of JAK–STAT, *Atg2* expression is reduced, large and irregular LDs are formed, and the bacteria survive and proliferate within the immune cell. However, if the JAK–STAT pathway is inhibited, *Atg2* expression is increased and ATG2 protein is found surrounding LDs and bacteria (red dots). The LDs maintain small and circular structures and the bacteria are less able to survive or replicate.

Our data suggest a model in which STAT-driven *Atg2* repression deregulates lipid storage upon infection to the advantage of mycobacteria (Fig. 6). Despite potent bactericidal mechanisms, macrophages are often unable to eradicate *M. tuberculosis*, which then persists in the organs of the infected individual^50^. Therefore, novel strategies are needed to counteract bacterial latency and permit killing. Our work suggests two strategies to target mycobacterial infection by altering lipid metabolism in macrophages. One such strategy is direct inhibition of DGAT1. The fact that ATG2A overexpression is sufficient to reduce intracellular triglyceride in cultured THP-1 cells, and that *Atg2* overexpression is sufficient to reduce mycobacterial survival in fly cells, suggests another such strategy. The strong and specific repression of *Atg2* by JAK–STAT signalling, at least in *Drosophila*, indicates one regulatory mechanism for this gene, but it is clear that even in this comparatively simple system other regulators are critical. Identification of these other regulatory mechanisms may open new avenues for antimycobacterial therapies.

## Methods

### *Drosophila* stocks

The following stocks were used: from the Bloomington *Drosophila* Stock Center: *os*^*s*^ (BDSC 79), *Gal80*^*ts*^ (BDSC 7017, 7018, 7019, 7108), c564 (BDSC 6982), *HmlΔ*-Gal4 (BDSC 30140) and UAS-*Stat92E*-IR (BDSC 26899); from the Vienna *Drosophila* Resource Center: UAS-*upd3*-IR (VDRC 106869) and UAS-*Stat92E*-IR (VDRC 106980); from Hervé Agaisse (Yale University): UAS-*upd3*-IR; from Nathalie Franc (The Scripps Research Institute): *crq-*Gal4; from James Castelli-Gair Hombría (Centro Andaluz de Biología del Desarrollo): UAS-*dome*^*Δcyt*^.

### Survival analysis

Mycobacterial and *Drosophila* culture and infections were carried out substantially as described, using 5- to 9-day-old male flies^12^. Injected flies were incubated 20 flies to a vial and the number dead was recorded twice a day; all survival experiments were repeated at least three times with qualitatively similar results. When using a tubulin-*Gal80*^*ts*^ construct, crosses were kept at 18 °C and flies were collected and switched to 29 °C the day after eclosion; they were then maintained at 29 °C for the duration of the experiment. For experiments not involving tub-Gal80^ts^, flies were kept at 25 °C at all times.

### Intravital imaging

For haemocyte counts, 5–7-day-old flies carrying *HmlΔ*-Gal4, UAS-2xeGFP or *Crq*-Gal4, UAS-2xeGFP were injected with 500 colony-forming unit (CFU) *M. marinum* and imaged at indicated times. Flies were glued to coverslips and imaged immediately using a confocal microscope (SP5, Leica) equipped with an environmental control chamber. The environmental chamber was set to 25 °C or 29 °C when tub-Gal80^ts^ flies were used. Flies that died during the procedure were excluded from the analysis. GFP was excited at 488 nm with an argon laser and 5.63 μm sections were imaged through the coverslip, glue and dorsal cuticle. Flies carrying *HmlΔ*-dsRed.nls and *upd3*-Gal4, UAS-GFP were imaged 48 h after injection of 5,000 CFUs using the same procedure with sequential excitation at 488 and 561 nm, and with images taken every 0.69 μm.

### *Drosophila* cell culture and infection

S2R^+^ cells (DGRC, Bloomington, Indiana) were maintained in M3 medium supplemented with 10% fetal bovine serum and 50 U ml^−1^ penicillin–streptomycin. S2R^+^ cells were transfected using Effectene (Qiagen); 24 h later, they were washed and then infected with *M. marinum* (suspended in M3 medium supplemented with 10% fetal bovine serum and 1% penicillin–streptomycin) at a multiplicity of infection (MOI) of 10–20. Samples were collected after 24 or 48 h for western blotting, qRT–PCR or imaging.

For overexpression experiments, GFP-tagged human *ATG2A* (from plasmid pEGFP-C1-hAtg2A, Addgene, deposited by Noboru Mizushima) and *Drosophila upd3* (by PCR from *Drosophila* complementary DNA) were cloned into pPAC-HA, a vector containing the *Drosophila Actin 5C* promoter (from Nic Tapon (Francis Crick Institute)). In all overexpression experiments, the displayed control is cells transfected in parallel with the empty vector, in which a 3xHA peptide is expressed.

Phagocytosis assays on cultured cells were done as described^51^.

For RNAi experiments, cells were bathed in double-stranded RNA (dsRNA) targeting *Stat92E* or, as a control, *Renilla luciferase*. RNAi constructs were designed using the SnapDragon web tool with no predicted off-targets; template amplicons were produced by PCR from plasmid or *Drosophila* genomic DNA with the following primers: T7-RLucL, 5′-TAA TAC GAC TCA CTA TAG GGA GAC TGA TCA AGA GCG AAG AGG G-3′; T7-RLucR, 5′-TAA TAC GAC TCA CTA TAG GGA GAC TTT CAC GAA CTC GGT GTT G-3′; T7-Stat92eL, 5′-TAA TAC GAC TCA CTA TAG GGA GAA AGC TGC TTG CCC AAA ACT A-3′; T7-Stat92eR, 5′-TAA TAC GAC TCA CTA TAG GGA GAG TCG ACG ATA AAG GCA GAG C-3′; T7-mdyL, 5′-TAA TAC GAC TCA CTA TAG GGA GAG CGA CTT CTT AAA CTT GCG G-3′; and T7-mdyR, 5′-TAA TAC GAC TCA CTA TAG GGA GAG ATA TGG CCG ATA GGG GAA T-3′. dsRNA was made with the MEGAscript T7 kit (Ambion). For efficient knockdown, 1.2 μg dsRNA was added per well in 48-well plates containing 200,000–500,000 cells according to the DRSC RNAi protocol. After 3 days of dsRNA treatment, *M. marinum* was added to the dsRNA solution, at an MOI of 20–60 based on the initial number of cells added in the experiment. Cells were lysed in TRIzol 24–48 h post infection.

### Flow cytometry of *Drosophila* haemocytes and S2R+ cells

For cytotoxicity assays in uninfected and infected *Drosophila* S2R+ cells, the supernatant medium containing non-adherent cells was collected 24 h post infection and transferred directly into a FACS tube. The adherent S2R+ cells of the same sample were detached using 2 ml of ice-cold culture medium and pipetting up and down. The medium was then added to the previously collected supernatant sample. All samples were filtered with a 70 μm cell strainer filter (BD Biosciences) and kept on ice before FACS analysis. Five minutes before analysis, 5 μl of propidium iodide (PI) was added to 500 μl of samples to stain dead cells. Cells were analysed with a FACS Aria II (Becton Dickinson (BD)), using a 100 μm nozzle and the FACS-Diva software. All experiments included the following controls: *M. marinum* only samples with and without PI staining, and non-infected cells with and without PI.

For phagocytosis assays with phrodo-coated beads in uninfected and infected *Drosophila* S2R+ cells, supernatant and adherent cells were collected as described above 48 h post transfection. Cells (500,000) were spun down for 3 min at 21 °C, 1,400 r.p.m. and resuspended in fresh medium. pHrodo-coated beads were added at a concentration of 1/100 v/v and incubated with cells for 30 min at 25 °C. Cells were then spun for 3 min at 21 °C, 1,400 r.p.m. to remove free beads. Fresh medium was added and cells were incubated one more hour before analysis by FACS (BD LRS Fortessa). Samples were analysed in the ultraviolet channel (IndoViolet beads) and the Texas red channel (pHrodo). The experiment included the following controls: cells without beads and beads-only control (no pHrodo).

For measurement of *M. marinum* uptake by haemocytes *in vivo*, the different genotypes (HmlΔeGFP/+ and HmlΔeGFP>upd3-IR) were injected with *M. marinum* expressing Tomato fluorescent protein. Sixteen hours post infection 30–40 flies per sample were anaesthetized with CO_2_. The flies were carefully transferred onto a 70 μm mesh, and smashed and filtered in 10 ml ice-cold 1 × PBS with 2 mM EDTA. The cell suspension was centrifuged at 150 g for 10 min at 4 °C and the supernatant carefully removed. The cell pellet was washed in 10 ml 1xPBS with 2 mM EDTA twice, afterwards the cells were resuspended in 500 μl of 1 × PBS with 2 mM EDTA and transferred to a FACS tube by filtering the solution through a 70 μm filter mesh. Samples were acquired with a BD Fortessa and analysed using the FlowJo analysis software. Four samples per genotype were analysed.

### *In vitro* mycobacterial infection assays

Luminescent reporter strain infection: a validated luminescent reporter strain of *M. bovis* BCG (BCG-*lux*) encoding the Vibrio *lux AB* gene, generated as described^52^, was used to infect macrophages. Correlation between CFUs and luminescence was confirmed before experiments. Primary monocyte-derived human macrophages, generated (as previously described^53^) from healthy consented subjects (Regional Ethics approval: REC: 12/WA/0148 and 4/EE/1187) or THP-1 cells (ATCC), were differentiated by treatment with 5 ng ml^−1^ PMA 48 h before infection, inoculated with BCG-*lux* (at an MOI of 5:1) for 2 h at 37 °C, repeatedly washed, then incubated for 24 h at 37 °C in the presence of compounds as indicated. Unless otherwise stated, recombinant cytokines (Peprotech) were used at 20 ng ml^−1^ and added to cells following 2 h incubation with mycobacteria. Where indicated, cells were pretreated with DGAT1 inhibitor T863 (10 μM) for 48 h and then 24 h post infection, IL-6 was treated for 24 h post infection at 80 ng μl^−1^. Cells were lysed and luminescence measured as described^53^. Experiments were carried out in sextuplicate. Results are representative of at least three separate experiments.

*M. tuberculosis* infection: *M. tuberculosis* ΔleuD ΔpanCD (Bleupan)^54^ stably transfected with pMSP12::GFP was grown in Middlebrook 7H9 broth (Difco) containing 0.5% glycerol, 0.05% Tween 80, 10% OADC (BD), 0.05 mg ml^−1^ L-leucine, 0.024 mg ml^−1^ calcium pantothenate and 0.2 mg ml^−1^ Hygromycin B in 250 ml sterile disposable Erlenmeyer flasks with mild agitation at 37 °C. Bacterial cells were harvested at mid log phase by centrifugation at 3,000 *g* for 7 min, pellets were resuspended in 7H9 broth containing leucine and pantothenate supplements as above plus 18% glycerol and frozen at −80 °C until use. Cells were infected at an MOI of 5:1, lysed at times indicated and CFUs enumerated as described^55^.

### Confocal immunofluorescence on human cells

Primary human macrophages were grown on glass coverslips, infected with *M. bovis* BCG (BCG-*lux*) and treated as described, rinsed with PBS, fixed with methanol and permeabilized with 0.1% Triton X-100 (Sigma)^56^ before being stained with Nile red 1:10,000 (dissolved in isopropanol), rinsed with water and then mounted with ProLong Gold antifade containing DAPI (Invitrogen). Images were acquired on a Zeiss LSM880 confocal microscope (Plan-Apochromat × 63/1.40 Oil-immersion lens) and analysed with Zen 2010 (Carl Zeiss), and fluorescence per cell measured by Volocity software.

### Antibodies and western blots

For the anti-ATG2A blotting shown in Fig. 5, primary human macrophages were treated with IL-6 80 ng μl^−1^ for 24 h before being lysed. The primary antibody used was anti-ATG2A pAb (MBL Life Science) at 1:400. Other western blots were performed with anti-*Drosophila* Atg8 (courtesy Katja Köhler (University of Zürich)^57^ and G. Juhasz (Eötvös Loránd University)^58^, both used at 1:200) and anti-α-tubulin (Developmental Studies Hybridoma Bank 12G10, used 1:10,000). Immunofluorescence on fly cells was performed with fluorescein isothiocyanate-labelled anti-*M tuberculosis* (Invitrogen PA1-28997, used 1:50) and rabbit anti-GFP (Invitrogen A-11122, used 1:100). Immunofluorescence on human cells was performed with anti-V5 from Novus Biologicals (NB 600-381), 1:400.

### *M. marinum* culture

*M. marinum* strain M and fluorescent derivatives were grown standing at 25° in Middlebrook 7H9 media, supplemented with OADC, Tween-80, glycerol and antibiotics as necessary to maintain fluorescent plasmids^12^. Single-cell bacterial suspensions were produced as follows. Cultures were pelleted by centrifugation at 4,000 *g* for 5 min, then resuspended in PBS+0.2% Tween-80. Clumps were then separated by centrifugation at 200 *g* for 5 min. The supernatant from this centrifugation contained primarily single bacterial cells. *M. marinum* strains were all strain M carrying various fluorescent proteins under the control of the *msp12* promoter^59^; all were courtesy of Lalita Ramakrishnan and Antonio Pagan (University of Cambridge).

### Statistics

Statistical analyses were performed using GraphPad Prism or R. Two-way analysis of variance, Mann–Whitney test and Kolmogorov–Smirnov test were used in this study. Statistical analysis was only performed on experiments with a least four samples per condition. *P*-values are as follows: *P<0.05, **P<0.01 and ***P<0.001. Unless otherwise noted, error bars correspond to s.e.m.

### qRT–PCR analysis

RNA was extracted from adult flies using TRIzol (Invitrogen) according to the manufacturer's directions, except that samples from which mycobacterial RNA was amplified were first homogenized in a 3:1 mixture of chloroform and methanol^60^. Mycobacterial quantification was performed using a standard in which known quantities of fly and bacteria were mixed and then treated the same as injected samples. qRT–PCR was performed as described^10^,^61^. PCR primers used in this work are listed in Supplementary Table 1.

### Data availability

All relevant data are available from the authors, except where precluded by human subject confidentiality.

## Additional information

**How to cite this article:** Péan, C. B. *et al*. Regulation of phagocyte triglyceride by a STAT-ATG2 pathway controls mycobacterial infection. *Nat. Commun.* **8**, 14642 doi: 10.1038/ncomms14642 (2017).

**Publisher's note:** Springer Nature remains neutral with regard to jurisdictional claims in published maps and institutional affiliations.

## Supplementary Material

Supplementary Information Supplementary Figures and Supplementary Table
