# Supplementary material for: Regulation of phagocyte triglyceride by a STAT-ATG2 pathway controls mycobacterial infection
Source: Nat Commun. 2017 Mar 6;8:14642. doi: 10.1038/ncomms14642 (PMC5343520; doi:10.1038/ncomms14642)
Supplement: Supplementary Information — Supplementary Figures and Supplementary Table [file ncomms14642-s1.pdf]

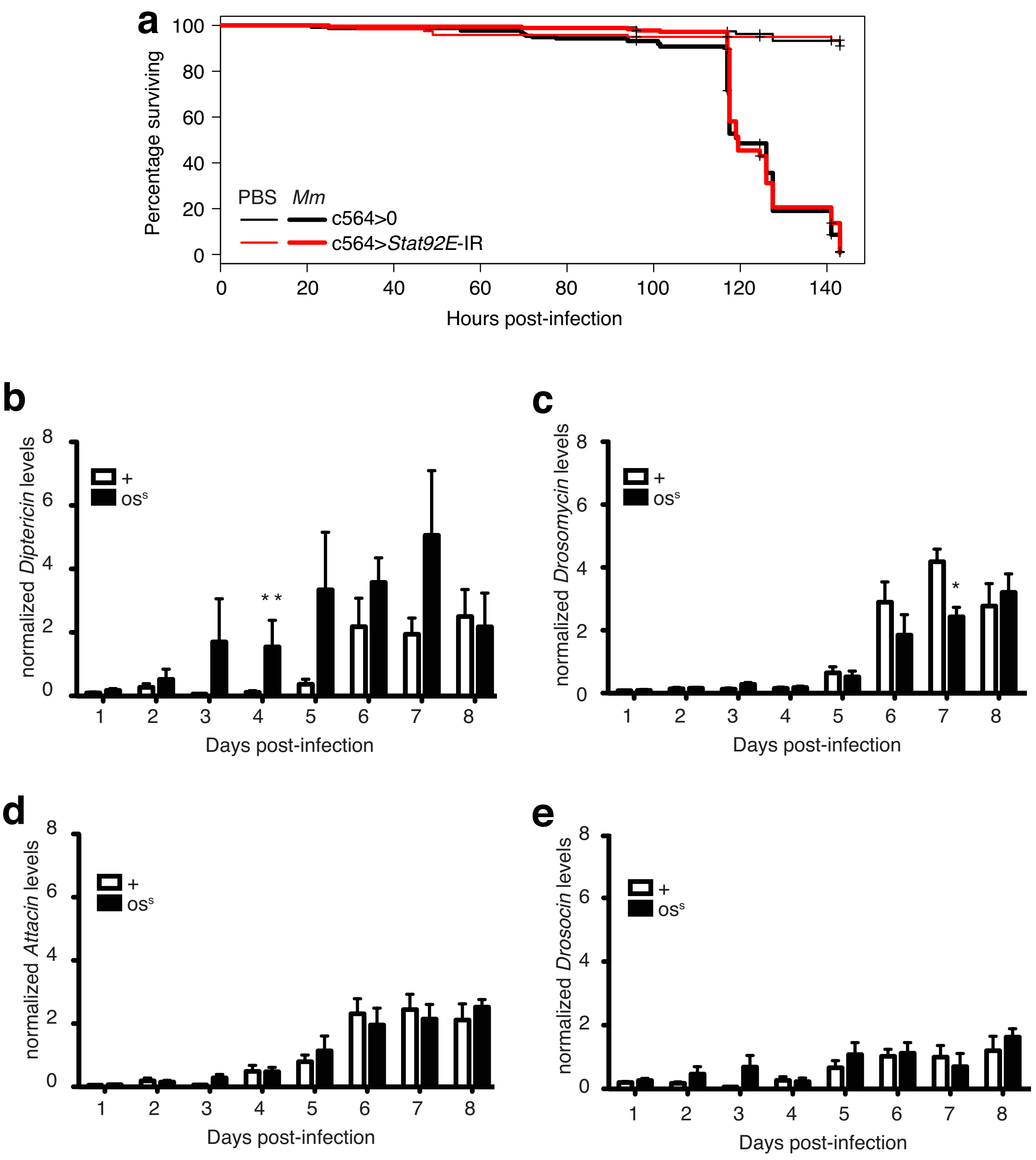

Supplementary Figure 1.

(a) Survival of flies with *Stat92E* knocked down in fat body (c564>*Stat92E*-IR) and controls (c564>0) after infection with 5000 CFU *M. marinum*.

(b-e) Normalised *Diptericin*, *Drosomycin*, *Attacin* and *Drosocin* expression over RpL1 in os^s mutants and controls at different timepoints following infection with *M.marinum* (500 CFUs). Only a few values are statistically different (*p<0.05 and **p<0.01 by Mann-Whitney test, n=6).

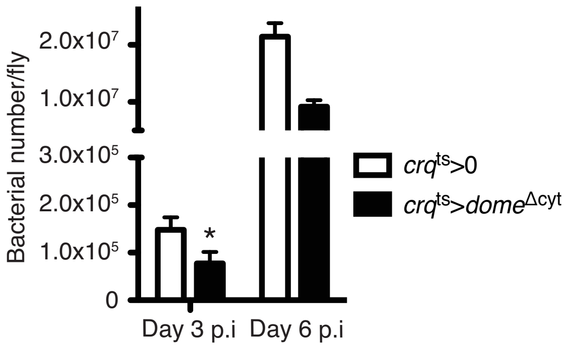

Supplementary Figure 2.

Numbers of *M marinum* at different times after infection in control (*w^1118^* ; *tub-Gal80^ts^* / + ; *crq-Gal4* / +) and inducible hemocyte-specific dominant negative *Domeless* (*w^1118^* ; *tub-Gal80^ts^* / + ; *crq-Gal4* / *UAS-DomeΔcyt-3-1*) flies, assayed by qRT-PCR. Values are statistically different at day 3 and 6 after infection (*$p<0.05$ and **$p<0.01$ by Mann-Whitney test, n=6).

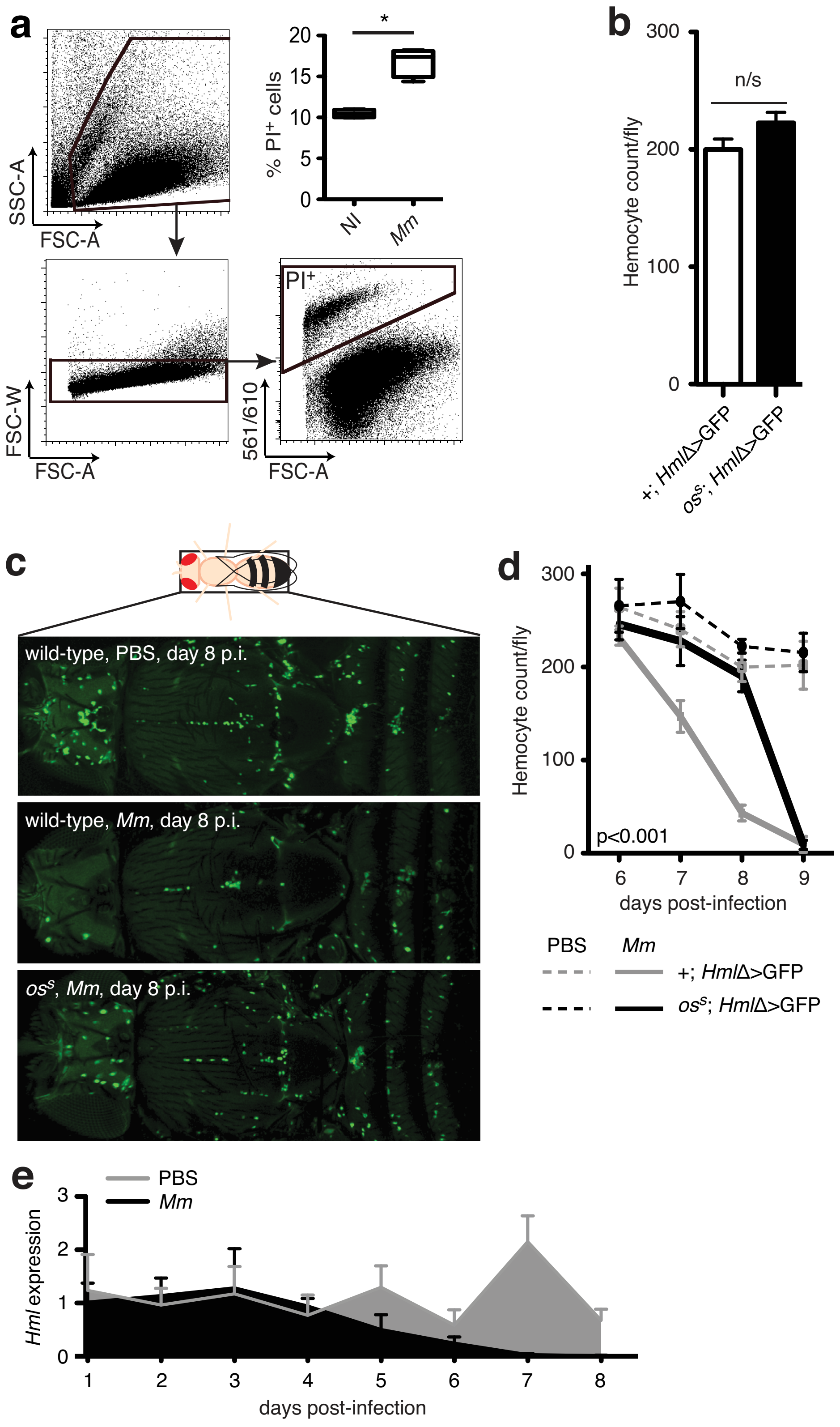

Supplementary Figure 3.

(a) FACS scheme for analysis of cell death in S2R+ cells (exclusion of debris followed by exclusion of doublets based on light-scattering characteristics, followed by quantification of cell death based on PI incorporation). Upper right corner, percentage of dead (PI^+) $S2R^+$ cells 24 hours after infection with *M. marinum* (MOI=20) or not infected. Values are statistically different (*, $p<0.05$) by Mann-Whitney test, n=9.

(b) Number of GFP^+ cells in 400μm sections in the back of flies carrying *HmlΔ*-Gal4 driving UAS-GFP on the *os*^{s} background or the isogenic wild-type control. Intravital imaging was performed on

adult flies from 5 days after hatching and analysed with the imaging software Imaris. Values are not statistically different by Mann-Whitney test, n=57 (wild-type)/56 (*os*^{s}).
(c) Number of GFP^{+} cells at different times after infection with *M. marinum* at 500CFUs or post-injection with PBS. Fly genotypes and imaging technique as in b. Representative images at day 8 post-infection or PBS-injected are shown.
(d) Quantification of hemocyte numbers, taken from images as shown in c. for all genotypes (left). P-value for difference between *os*^{s} and control curves by two-way ANOVA, n=4/5 flies per timepoint except for day 9 where n=2/3.
(e) *Hemolectin* expression in wild-type flies at different times after injection with *M. marinum* (500 CFUs) or PBS. *Hml* expression was assayed by qRT-PCR and normalized to *Rpl1*.

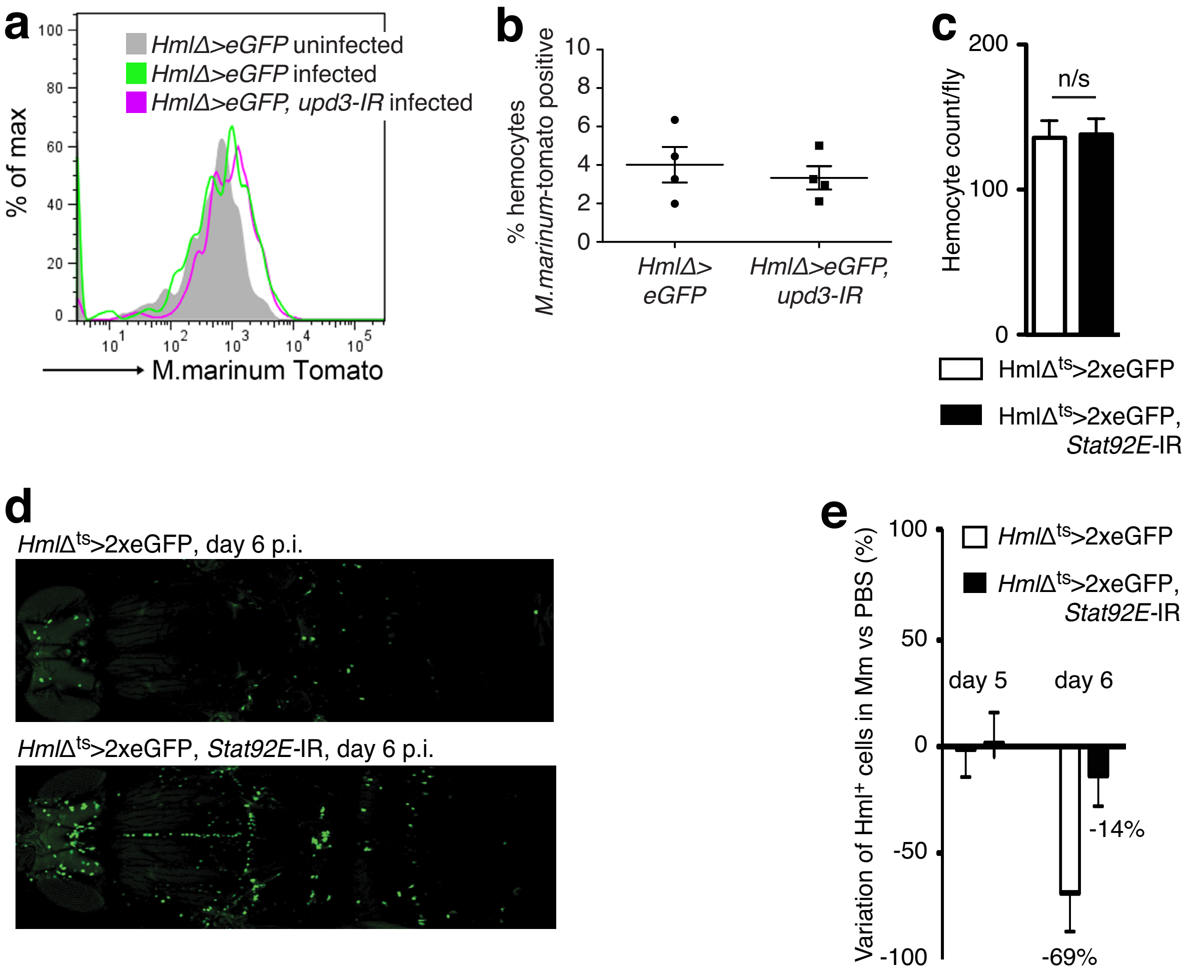

Supplementary Figure 4.
(a) Histogram showing tomato fluorescence in hemocytes sorted using *HmlΔ-Gal4* driven eGFP from flies uninfected or infected with tomato-expressing *M. marinum* (*msp12::tomato*).
(b) Quantification of the tomato-positive cell fraction from (a).
(c) Number of GFP$^+$ cells in 400μm sections in the back of flies carrying *HmlΔ-Gal4* driving *UAS-GFP* and the inducible-hemocyte-specific *Stat92E*-knockdown (w^{1118}, UAS-*Stat92E*-IR; UAS-*Stat92E*-IR / *HmlΔ-Gal4, UAS-2xeGFP* ; tub-Gal80^{ts} / +) or in control flies (w^{1118}; *HmlΔ-Gal4, UAS-2xeGFP* / + ; tub-Gal80^{ts} / +). Flies were switched from 18° to 29° immediately after hatching and kept at that temperature for 5 to 7 days prior to imaging. Values are not statistically different, n=8.
(d) Representative images of GFP$^+$ cells in live flies after 6 days of infection. Infectious dose was 500 CFU, genotypes and temperature as in (a).
(e) Variation in the number of GFP$^+$ cells between *M. marinum* and PBS-injected flies, for each genotype as described in e (quantification from images as in (b)). Values are statistically different after 5 days of infection (**, p<0.01 by Mann-Whitney test, n=4/5 per timepoint).

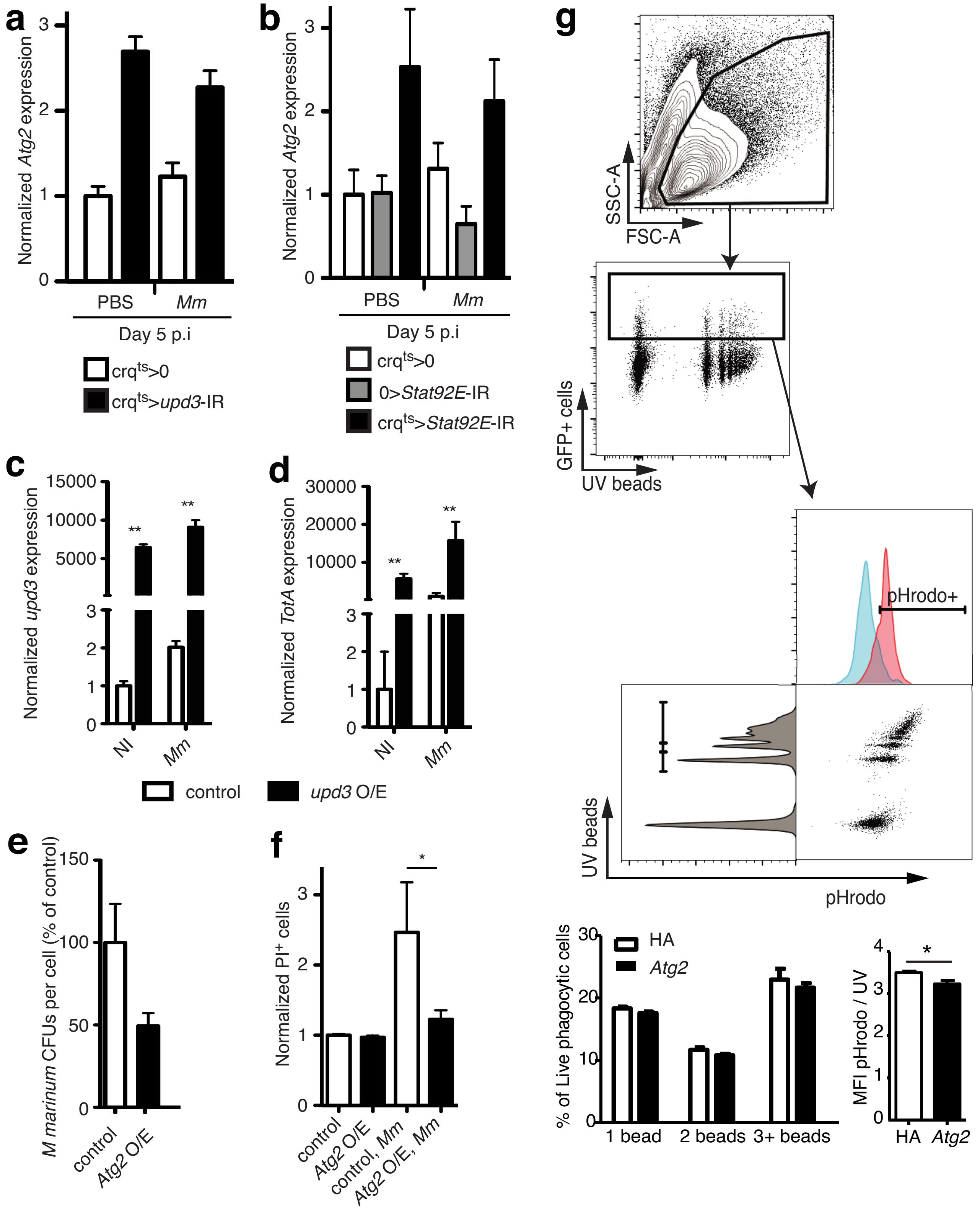

Supplementary Figure 5.

(a) Expression of *Atg2* by qRT-PCR in control (w^{1118}; *tub-Gal80^{ts}* / + ; *crq-Gal4* / +) and inducible hemocyte-specific *upd3*-knockdown *(w^{1118}; UAS-upd3-IR / tub-Gal80^{ts} ; crq-Gal4* / +) flies 5 days after *M. marinum* infection or PBS injection. Normalized to *Rpl1*. Values for PBS or Mm injected flies are statistically different (*, p<0.05 by Mann-Whitney test, n=5).

(b) Expression of *Atg2* by qRT-PCR in control (w^{1118}; *tub-Gal80^{ts}* / + ; *crq-Gal4* / +) and inducible hemocyte-specific *Stat92E*-knockdown *(w^{1118}, UAS-Stat92E-IR; UAS-Stat92E-IR / tub-Gal80^{ts} ; crq-Gal4* / +) flies 5 days after *M. marinum* infection or PBS injection. Normalized to *Rpl1*.

(c) Expression of *upd3* by qRT-PCR in infected or uninfected S2R$^+$ cells overexpressing *HA* (control) or *upd3*. Values are statistically different (**p<0.01 by Mann-Whitney test, n=6).
(d) For the same samples as in (a), TotA expression was assayed by qRT-PCR. Values are statistically different (**p<0.01 by Mann-Whitney test, n=6).
(e) Viable intracellular *M. marinum* in S2R$^+$ cells overexpressing *HA* (control) or *Atg2*. Bacterial count was assayed by plating lysed S2R$^+$ cells after 24 hours of infection and colony forming units were assessed after a 2-week incubation at 29 degrees, n=3.
(f) Cell death in control and *Atg2*-overexpressing cells infected with *M. marinum* or uninfected, quantified as in Figure 2A. Values for *Mm*-infected cells are statistically different (*, p<0.05 by Mann-Whitney test, n=3 for *Atg2* O/E and n=9 for controls).
(g) A pulse-chase phagocytosis experiment using pHrodo™-coated UV beads or UV beads alone on S2R$^+$ cells overexpressing GFP-ATG2 or control cells. FACS analysis of the cells after the chase period is presented on the left. For cells overexpressing GFP-ATG2, GFP$^+$ cells were selected for the analysis. The percentage of live cells internalizing one, two or more than two beads was assessed by quantifying pHrodo$^+$ cells at increasing UV intensities (top right). To distinguish differential acidification of the endosomes between the two genotypes, the Mean Fluorescence Intensity of pHrodo in cells cultured with pHrodo-coated UV beads over the same cells cultured with UVbeads alone was measured (bottom right). Values for the percentage of live phagocytic cells are not statistically different by Mann-Whitney test, n=6. For the mean pHrodo/UV, values are statistically significant (*p<0.05 by Mann Whitney test, n=12).

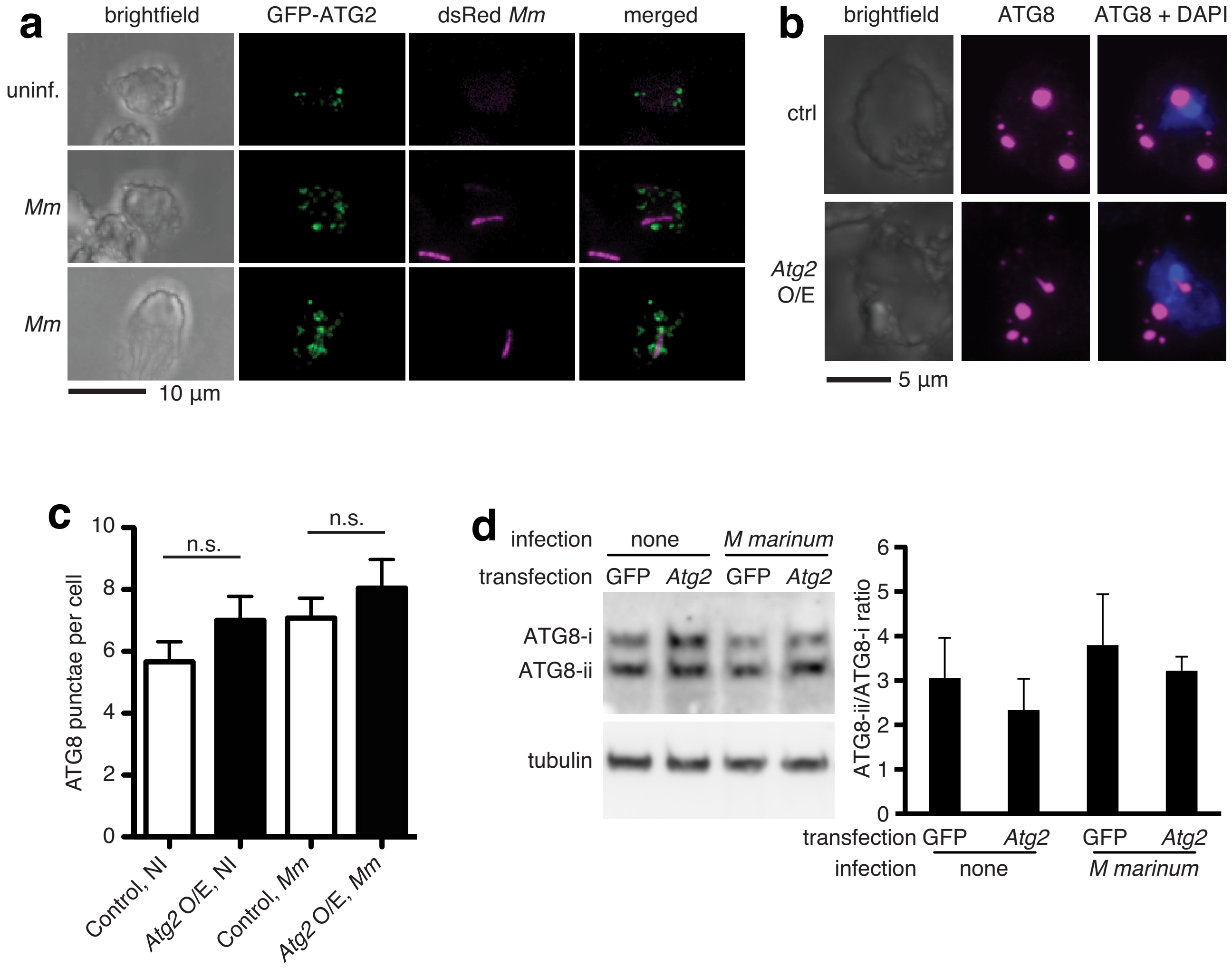

Supplementary Figure 6.

(a) Typical images showing localization of GFP-ATG2 in S2R$^+$ cells without (top row) or with (lower two rows) infection by dsRed-expressing *M. marinum*. The middle row shows intracellular *M. marinum* without any evident colocalized ATG2-GFP; the bottom row shows an ATG2-coated intracellular bacterium.

(b) Typical images showing ATG8 punctae (magenta) in control and *Atg2*-overexpressing cells. For this experiment, a rabbit anti-Atg8 Antibody (Cherry Lab) was used in combination with an anti-rabbit Cy5 secondary antibody (Jackson Laboratories).

(c) Quantification of ATG8 puncta numbers from cells treated as in (b). Values are not statistically different (Mann-Whitney test, n=48 to 65).

(d) Relative levels of ATG8-I, ATG8-II, and α-tubulin in control cells and cells overexpressing *Atg2* and treated with BafA1. For this experiment, a rabbit anti-ATG8 Antibody (Köhler Lab) was used in combination with an anti-rabbit HRP secondary antibody (Invitrogen). The quantification (right) reveals no difference in ATG8-II between cells overexpressing *Atg2* and controls, n=3. Full scanned areas for the lanes shown are in Supplementary Figure 7.

ATG8-i
ATG8-ii

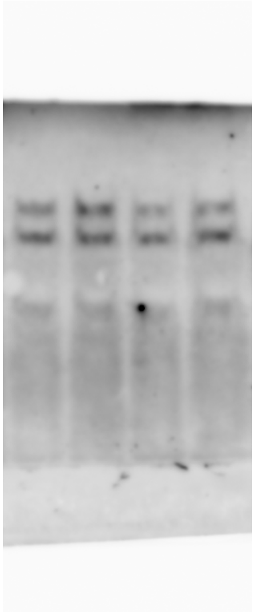

tubulin

GFP (transfection control)

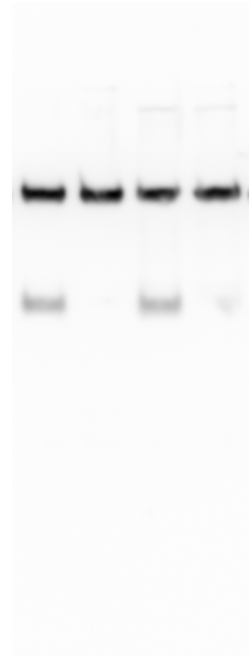

Supplementary Figure 7.
Full scanned areas for the blots shown in Supplementary Figure 6. The tubulin blot was probed simultaneously with anti-GFP to detect the control transfection (GFP alone) and the experimental transfection (ATG2-GFP). The ATG2-GFP band cannot be seen because this portion of the blot was not imaged (the ~250kDa ATG2-GFP fusion protein resolves poorly on the 13% gel used here).

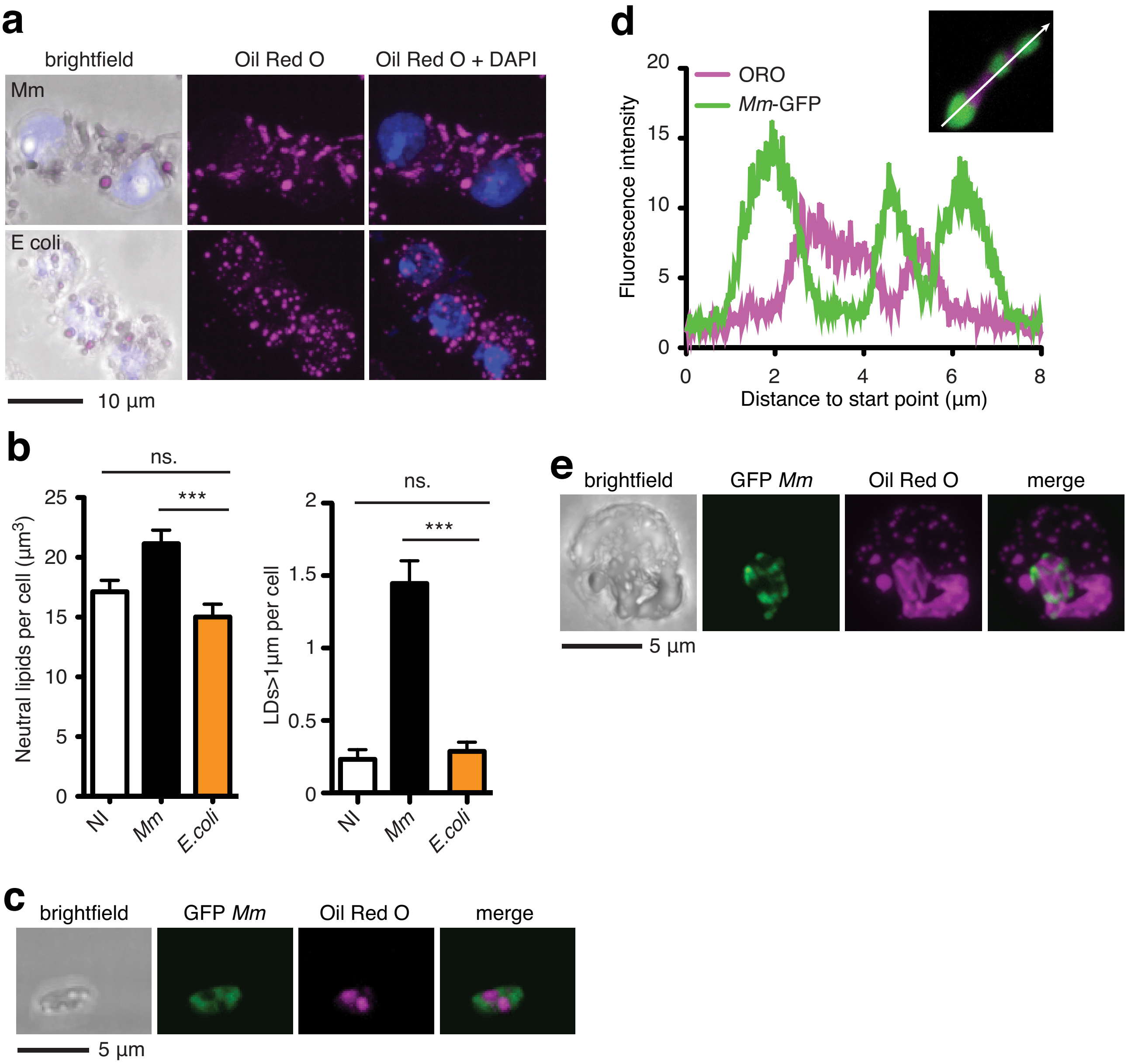

Supplementary Figure 8.
(a) Oil Red O-stained neutral lipids in cells infected with *M. marinum* or *E. coli* (MOI 10).
(b) Quantification of lipid volume per cell and total number of lipid droplets with at least one axis >1µm in length. Values are not statistically different between *E. coli* infected cells and non-infected cells. When comparing *Mm*-infected cells and *E. coli* infected cells, values are statistically different (***, $p<0.001$) by Mann-Whitney test. n=130 for non-infected cells, n=110 for *M.m.*-infected cells, and n=94 for *E. coli* infected cells.
(c) Extracellular GFP-expressing *M. marinum,* stained with Oil Red O.
(d) Fluorescence localization and intensity in a bacterium treated as in (C) to show non-overlap between GFP-Mm and ORO within *M. marinum*.
(e) Localization of intracellular GFP-expressing *M. marinum* and Oil Red O-stained neutral lipid. Though the bacteria are associated with a large, irregular lipid inclusion, most of the lipids are not within the bacteria.

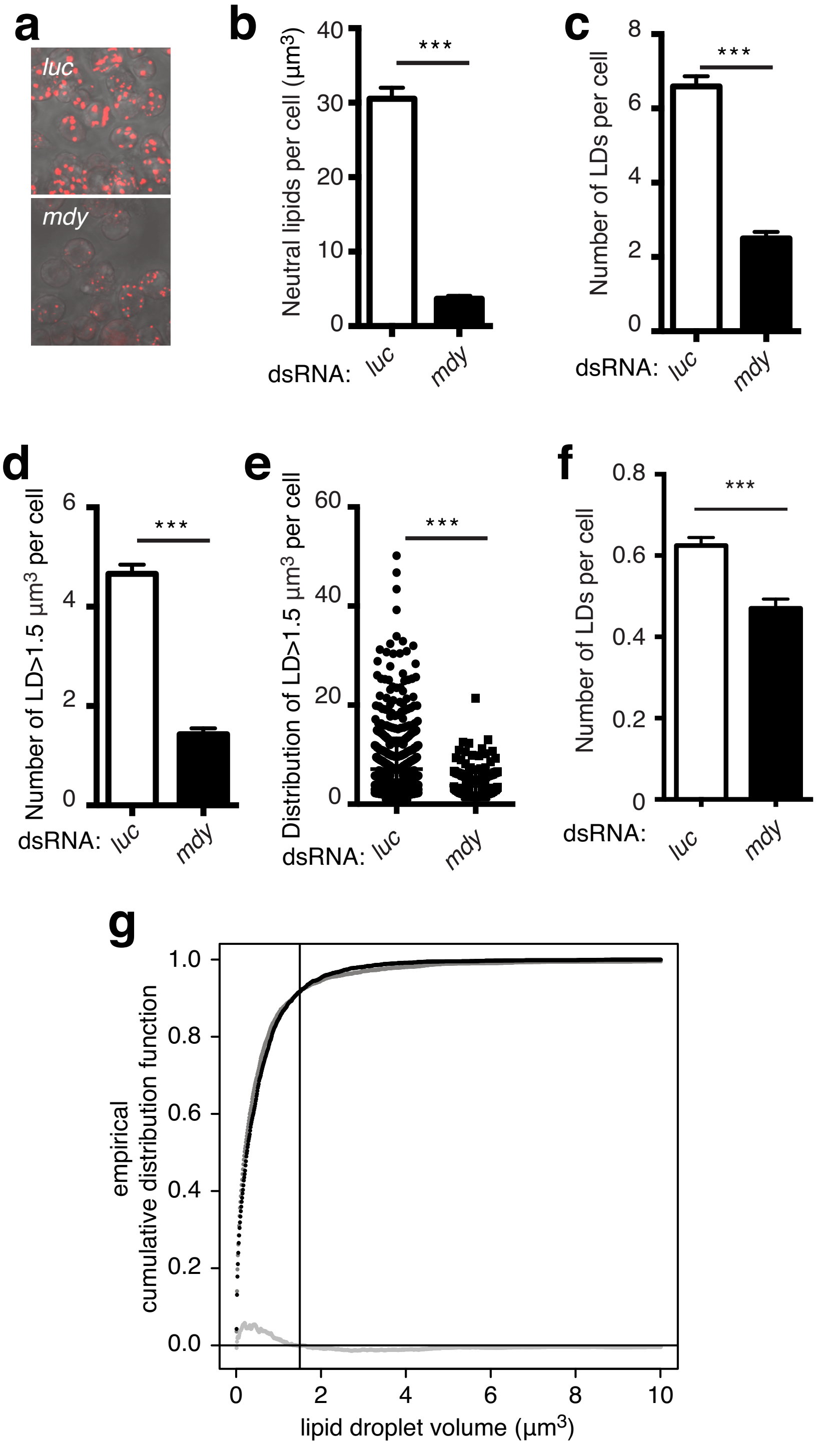

Supplementary Figure 9.
(a) BODIPY 500/510 staining of S2R^{+} cells showing reduced amount of lipid droplets in cells treated for 3 days with *mdy* dsRNA or *luc* dsRNA.
(b) Quantification of neutral lipid volume per cell in *mdy* and *luc* dsRNA treated cells. Values are statistically different (***, p<0.001) by Mann-Whitney test, n=232 for *mdy* dsRNA, n=244 for *Luc* dsRNA).
(c) Total LD (stained with BODIPY 500/510) per cell in S2R^{+} cells treated with either *mdy* dsRNA or *Luc* dsRNA as control. Values are statistically different (***, p<0.001) by Mann-Whitney test, n=232 for *mdy* dsRNA, n=244 for *Luc* dsRNA).
(d) Number of LDs (stained with BODIPY 500/510) of volume superior or equal to 1.5 µm ^{3} in cells treated with dsRNA for *mdy* or *Luc*. Values are statistically different (***, p<0.001) by Mann-Whitney test, n=232 for *mdy* dsRNA, n=244 for *Luc* dsRNA).

(e) Distribution of lipid droplets of volume superior or equal to 1.5 μm^3 in cells treated with dsRNA for *mdy* or *Luc*. Volumes less than 1.5 μm^3 are not shown. Values are statistically different (***, p<0.001) by Mann-Whitney test, n=200 for *mdy* dsRNA, n=856 for *Luc* dsRNA).
(f) Total LD (stained with BODIPY 500/510) per cells in infected S2R$^+$ cells treated with either *mdy* dsRNA or *Luc* dsRNA. Values are statistically different (***, p<0.001) by Mann-Whitney test, n=141 for *mdy* dsRNA, n=193 for *Luc* dsRNA).
(g) Empirical cumulative distribution function (ECDF) of lipid droplet size in infected (black line, n=4050) versus non-infected cells (dark grey, n=4210). In light grey, the difference between non-infected minus infected. The two ECDF's cross at a volume of 1.5 μm^3.

Supplementary Table 1. RT-PCR primers used in this work.

Gene	**Forward primer**	**Reverse primer**
Attacin	CACAATGTGGTGGGTCAGG	GGCACCATGACCAGCATT
Atg1	GTCAGCCTGGTCATGGAGTAT	CGTCCCCTTGACACTCAGAT
Atg2	CAAAAGACCTGTACGCAGAGC	TACTGAGCAACAGGTTTCGTC
Atg3	GACAATGGAGGAGACAAAGGAA	GTCCATGTCAATAGCCTCGTC
Atg4a	TGGGTCCTGGGAAAGAAGTA	AATGTCCCGTCGGATGAG
Atg5	ACGCTATATAAGCGCCGAAC	CAGCAGATCGTATAGGACACCA
Atg6	CGAGCAGCTGGAGAAGATTAG	TGTATTGCCATTGTCTCCGTA
Atg7	AGGATGCGCTGTAGCTAGAAA	CGCTGTCCAAAAGGGTTATG
Atg9	TTCAACATTGACTTCATCTTGTTTC	CTGCCAATTGTTCTGGAAGG
Atg12	AATGGCAGAGACACCAGAATC	TGGCGTTCAGAAGGATACAA
Atg13	ACCTCGCTGGAGGACTACG	CGATGAGGAGCCCGTTAG
Atg14	GCGTCAGAAACGGAAACATT	TGTAGCGCTGCTGCTTCTC
Atg16	TGGACAGTGATGGACAATCG	GCTACCGGTCACCACTTTG
Atg17	CGACTATCTTAACAAGGACAAGTGC	CATTCAGATCGTAGGCAGAGC
Atg18a	CGTCAACTTCAACCAGAACAT	TTGTCCAGGGTCGAGTCC
Diptericin	ACCGCAGTACCCACTCAATC	CCCAAGTGCTGTCCATATCC
Drosocin	CCATCGAGGATCACCTGACT	CTTTAGGCGGGCAGAATG
Drosomycin	GTACTTGTTCGCCCTCTTCG	CTTGCACACACGACGACAG
Hemolectin	CGATGATGACGACGAGGATA	GGCTTTGAGGATGTTGAAGC
pyrG (M marinum)	ACCGCTACGAGGTCAACAAT	ATTCGACGAACTCCACCAAG
Rpl1	TCCACCTTGAAGAAGGGCTA	TTGCGGATCTCCTCAGACTT
Turandot A	CCAAAATGAATTCTTCAACTGC	GAATAGCCCATGCATAGAGGAC
Stat92E	ACTAGTGGAACACCGCATCA	AGTCCGCCATTCCAAAGTC
upd3	ACTGGGAGAACACCTGCAAT	GCCCGTTTGGTTCTGTAGAT
